# Strontium isotopes and concentrations in cremated bones suggest an increased salt consumption in Gallo-Roman diet

**DOI:** 10.1038/s41598-022-12880-4

**Published:** 2022-06-03

**Authors:** Sarah Dalle, Christophe Snoeck, Amanda Sengeløv, Kevin Salesse, Marta Hlad, Rica Annaert, Tom Boonants, Mathieu Boudin, Giacomo Capuzzo, Carina T. Gerritzen, Steven Goderis, Charlotte Sabaux, Elisavet Stamataki, Martine Vercauteren, Barbara Veselka, Eugène Warmenbol, Guy De Mulder

**Affiliations:** 1 Department of Archaeology, Ghent University, Sint-Pietersnieuwstraat 35, 9000 Ghent, Belgium; 2 Maritime Cultures Research Institute, Department of Art Sciences and Archaeology, Vrije Universiteit Brussel, Pleinlaan 2, 1050 Brussels, Belgium; 3 Analytical, Environmental and Geo-Chemistry, Department of Chemistry, Vrije Universiteit Brussel, Pleinlaan 2, 1050 Brussels, Belgium; 4 G-Time Laboratory, Department of Geosciences, Environment and Society (DGES), Université Libre de Bruxelles, CP160/02, Avenue F.D. Roosevelt 50, 1050 Brussels, Belgium; 5 Anthropology and Human Genetics, Department of Biology of Organisms and Ecology, Université Libre de Bruxelles, CP192, Avenue F.D. Roosevelt 50, 1050 Brussels, Belgium; 6 Department of Anthropology, Faculty of Science, Masaryk University, Kotlářská 2, 611 37 Brno, Czech Republic; 7 Flemish Heritage Agency, Havenlaan 88/5, 1000 Brussels, Belgium; 8 Radiocarbon Dating Lab, Royal Institute for Cultural Heritage, Jubelpark 1, 1000 Brussels, Belgium; 9 Centre de Recherches en Archéologie Et Patrimoine, Department of History, Arts, and Archaeology, Université Libre de Bruxelles, CP133, Avenue F.D. Roosevelt 50, 1050 Brussels, Belgium

**Keywords:** Geochemistry, Archaeology, Archaeology, Biological anthropology, Social anthropology

## Abstract

The high temperatures reached during cremation lead to the destruction of organic matter preventing the use of traditional isotopic methods for dietary reconstructions. Still, strontium isotope (^87^Sr/^86^Sr) and concentration ([Sr]) analyses of cremated human remains offer a novel way to assess changing consumption patterns in past populations that practiced cremation, as evidenced by a large amount of new data obtained from Metal Ages and Gallo-Roman human remains from Destelbergen, Belgium. The Gallo-Roman results show significantly higher [Sr] and a narrower interquartile range in ^87^Sr/^86^Sr (0.7093–0.7095), close to the value of modern-day seawater (0.7092). This contrasts with the Metal Ages results, which display lower concentrations and a wider range in ^87^Sr/^86^Sr (0.7094–0.7098). This typical Sr signature is also reflected in other sites and is most likely related to an introduction of marine Sr in the form of salt as a food preservative (e.g. salt-rich preserved meat, fish and fish sauce). Paradoxically, this study highlights caution is needed when using ^87^Sr/^86^Sr for palaeomobility studies in populations with high salt consumption.

## Introduction

**Figure 1:**
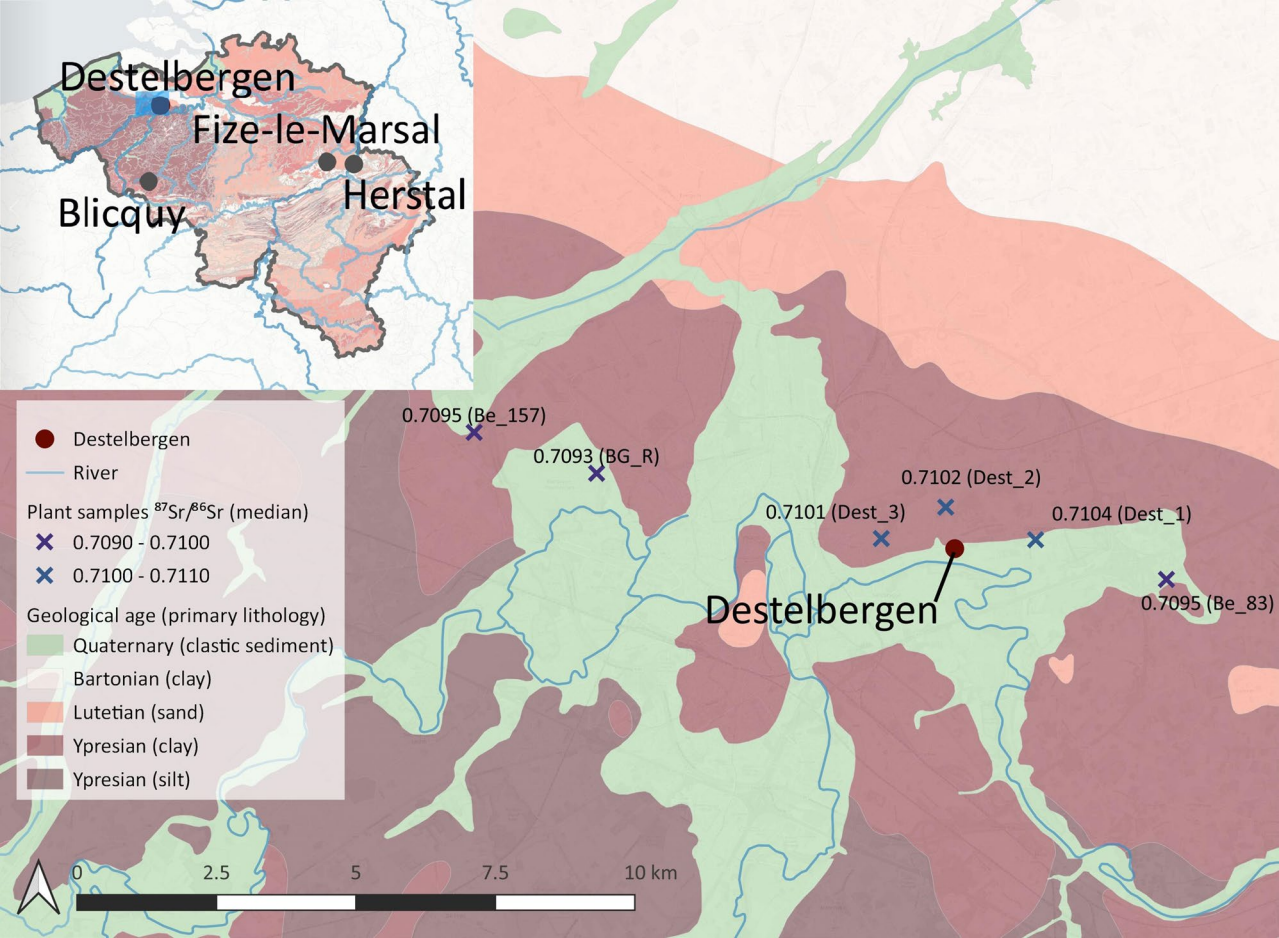
Location of the Destelbergen, Blicquy, Fize-le-Marsal and Herstal sites within Belgium on the inset map and the six new plant sampling locations around Destelbergen with median ^87^Sr/^86^Sr superimposed on a geological map (map created using QGIS version 3.12.0-București, https://qgis.org/).

The site of Destelbergen “Eenbeekeinde”, close to Ghent, Belgium, is located on the left bank of the Scheldt river (Fig. 1) and is one of the rare sites where a large number of both Metal Ages and Gallo-Roman cremation burials were excavated^1–4^. Steady advancements in Sr isotopic applications prove that cremated bone shows even less contamination than previously favoured tooth enamel^5^ and provide new methods to gain knowledge on communities who practiced cremation. As such, this site offers a unique opportunity to study shifts in mobility, diet and landscape use from the Iron Age to the Roman period by combining archaeological evidence with state-of-the-art ^87^Sr/^86^Sr and [Sr] analyses of cremated human remains. In the Metal Ages cemetery (107 burials), a general chronological evolution from east to west was found^6^. In the Gallo-Roman cemetery, which was with its 204 burials far more extensive than most common familial cemeteries of this period^7^, graves were usually simple pits. Besides Destelbergen, both new and published data from three other Belgian sites was analysed for comparison. Blicquy offered just like Destelbergen both Metal Ages and Roman cemeteries at close distance from each other. Additionally, in relatively close proximity to each other and located further inland in the Meuse basin, two more sites serve as reference data. Metal Ages cemetery Herstal “Pré Wigier” delivered already published results^8^. The cemetery Fize-le-Marsal “Beauflot-Pivache” revealed Gallo-Roman burials on which new analyses were performed. For more information on the sites, see Suppl. Text 1.

Sufficient evidence shows that in western Europe the Iron Age diet was fairly homogeneous, rich in animal protein in the form of meat and dairy and almost exclusively terrestrial e.g.:^9–11^. Although, for Belgium, clear archaeozoological and archaeobotanical evidence on Iron Age food economies is scarce, data from neighbouring areas indicates that the population relied mainly on varying proportions of cattle, sheep, and pig as main suppliers of animal protein, and cereal and, to a lesser extent, pulses as a staple food^10,12,13^. A total lack of fish bone at Iron Age sites led to the conclusion that fish was not consumed in Belgium, nor in England, but was yet present in some areas such as in Scotland and the Netherlands^14^. Generally, wild resources, such as game and fish, were not prominent in the protohistoric diet in western Europe^12^ and are even suggested to have been taboo^14^.

The diet in the Roman period, however, is expected to have changed significantly under the important influence of the Mediterranean world. Still, cereal and some pulses remained the staple food^13,15^, with the specific dominant crop and livestock types largely following the local environmental demands^16^. Seeds and pollen in the Gallo-Roman wells in Destelbergen revealed the presence of nearby yards in which several Roman introductions were cultivated^4,17,18^. This shows a change in the amount and type of resources available and the implementation of Mediterranean influences in the diet^19^. In terms of livestock, in Destelbergen, the first century occupation was associated with some sheep bone while the settlement remains from the following centuries delivered mostly cattle with smaller amounts of pig and horse bone^2,4^.

Carbon (δ^13^C) and nitrogen (δ^15^N) isotope studies on cremated individuals are not informative for dietary studies because these isotopes are altered during cremation. The measurable δ^13^C after burning reflect the cremation conditions rather than the original dietary practices of the deceased during life^20–22^. Still, these analyses on inhumated human bone were able to paint an isotopic picture of Metal Ages and Roman diets. The Romano-British population generally revealed a significant rise in marine protein compared to the Metal Ages as attested by δ^13^C and δ^15^N studies^9,23–25^. Due to lacking studies on inhumated bone from Belgium, it is uncertain whether this shift can be extrapolated to Belgium. Fish sauce (a condiment made of salted, fermented fish) and molluscs are suggested as a potential source of this marine protein as these products are easier to transport and preserve than fresh fish^9,25,26^. In Belgium, the originally imported product from the Mediterranean seems to have been substituted by a locally produced fish sauce in the second and third centuries^27,28^, confirming an important local demand. Clearly, the consumption of marine food seems a Roman dietary addition in Belgium and England. In Destelbergen, however, no evidence of fish consumption has been uncovered, nor in the pottery, nor in the animal bone record. Absence of this archaeological evidence does not necessarily mean that these foods were not consumed. This lack of affirmation could be caused by the use of perishable (wooden), or else generic, unrecognised types of transport vessels^29^ and the fact that the applied excavation methods were not aimed at finding tiny fish bone fragments.

Another vital component in the diet for humans and domestic animals alike is salt^30^. Next to that and its multiple industrial uses, this mineral revolutionised food storage thanks to its antimicrobial properties, increased transportability, and availability of products such as meat (most often pork), fish and dairy (in butter and cheese) throughout the year^30–32^. Inscriptions from a submerged native Nehalennia temple in the Scheldt estuary (the Netherlands) indicate the presence of fish sauce and salt traders in the area^33,34^. Salt production at the North Sea coast goes back to at least Early Iron Age practices (seventh century BCE)^35^. This production is thought to have increased in the Roman period in part to sustain the military apparatus defending the northern borders of the Roman Empire, but in the process possibly markedly increasing the availability of salt in the region^7,35–37^. It remains, however, hard to assess to what degree the actual salt consumption in the wider population changed in the transition from Iron Age to Roman period. Van den Broeke sees an overall rise in the number of salt containers in the hinterland as a proxy for an increased salt consumption from the fourth century BCE onwards^38^. In Britain, intensification of salt production during the Late Iron Age has also been linked to the rise of meat preservation and in the trade of otherwise perishable foods^31,32^. The presence of salt container fragments in Destelbergen^4,35^ and many other settlement sites affirms the availability of salt in the Gallo-Roman economy. Isotopically, an increase in salted or brined food may have a significant impact on the ^87^Sr/^86^Sr and [Sr] of human bones and teeth^39,40^.

In the inland area with modest sandy ridges and wide river valleys, mixed subsistence farming including cattle, sheep and pig breeding in varying frequencies depending on the local environment is the expected form of economy during the Late Bronze Age and Early Iron Age^41^. A general cold episode with extreme wetness during the Early Iron Age (750–400 BCE) led to a shortening of the growing season for crops and contractions in settlement patterns^42^. In this period, cemeteries were usually not established adjacent to the settlement^43,44^. To date, indeed no indications for Metal Ages settlements have been found close to the Destelbergen cemetery. This fits in the concept of ‘wandering farmsteads’^41,45^. This paradigm encompasses that farms moved locations every other generation within a certain territory while maintaining an expanding central communal cemetery over generations. Especially in regions with fast degenerating sandy soils this moving of the farmstead to newly regenerated agricultural land is presumed necessary to keep crop production up to standards. Nevertheless, this model has been nuanced and longer continuity of settlements has been observed in more sustainable environments^46^ (Suppl. Text 2).

In contrast to the Destelbergen Metal Ages group, the Gallo-Roman group lived on site and as such provides a better idea of the settlement size and type. The number of burials (n = 204, based on fragmentary salvage excavations) covering an estimated period of 230 years (Flavian period until the end of third century^3^) in combination with the dense associated settlement remains suggest that the site in the Roman period was significantly larger than in the Metal Ages, needing an intensified agricultural production. The last two centuries BCE until around 400 CE experienced drier and warmer conditions, favourable for crop yields^42^. Recovered seeds and pollen suggest the inhabitants were essentially supporting themselves with staple foods from nearby fields, pastures, and vegetable plots^17^. The sandy region around Destelbergen, however, is not ideal for large surplus cereal productions to trade, possibly explaining the lack of villa domains and *vici*, but is expected to have been more suited for animal husbandry^34,47^. In well-connected places, the locally produced ‘farmer’s diet’ could be supplemented with imported, preservable special goods that could often not be produced locally, such as olive oil, wine, and salted products (e.g. salted meat and fish). Besides a clear consumption of local produce, the archaeobotanical evidence, next to considerable amounts of imported pottery^4^ and stone products^48^ in sites like Destelbergen, warns to not underestimate the extent of mobility of consumer goods during the Roman period; more than is reflected in the Metal Ages cemetery. Receiving traded goods, however, does not mean that the inhabitants themselves were more mobile^47^, but rather that there was a dynamic trade network.

During cremation, all organic matter is destroyed and the carbon present in the mineral fraction (often called bioapatite) is heavily altered, reflecting the cremation conditions rather than the diet^49,50^. As such, the only proxies currently available to investigate changes in mobility and potentially in diet in calcined human remains are ^87^Sr/^86^Sr and [Sr] as they are both unaltered by the cremation process and post-burial diagenesis^22,51^ (Suppl. Text 3, Suppl. Table 1a and 1b, Suppl. Fig. 2).

Strontium enters the food chain via plants and their ^87^Sr/^86^Sr vary depending on the type of underlying bedrock^52^. ^87^Sr/^86^Sr measured in human remains, therefore, reflect the geographical origin of the food and drinks consumed. Figure 1 shows a geological map of Destelbergen. The area is dominated by the Eocene Gentbrugge formation that is locally covered by 10 to 20 m of quaternary sediments of fluvial and aeolian origin^53^. Within a 15 km radius, several pre-quaternary lithologies occur, but are usually covered by similar quaternary sediments of varying thickness, potentially affecting the bioavailable strontium. As a result, it is difficult to predict the local bioavailable ^87^Sr/^86^Sr range without proper sampling of modern plants.

From the various food and drinks that can contribute to the Sr pool in bone and teeth, meat and milk do not contribute much as Sr accumulates mainly in the skeleton and not in soft tissue^52,54,55^. This also applies for marine organisms^56^. In contrast, plants (i.e. crops) represent the main dietary source of strontium^40,57^. Marine resources, especially salt, can also be a major contributor of Sr and heavily alter both ^87^Sr/^86^Sr and [Sr] in human remains^40,54,56,58,59^. This influence can easily be recognised since marine resources (and seawater) have a characteristic ^87^Sr/^86^Sr of 0.7092^60^. Demonstrating that salt can be impactful, a study on modern salted hams revealed that the meat took on the Sr isotopic signature of the applied salt^61^ and the [Sr] of several of the 16 hams raised up to 4.6 ppm^61^, instead of the usual low content in meat (pork fat and meat were found ranging from 0.1 to 1.1 ppm^54^). The 15 used marine and rock salts themselves ranged from 10 to 153 ppm and rock salts often have different ^87^Sr/^86^Sr than the current seawater value of 0.7092, depending on the age of the sea the evaporite mineral was formed in^61^. Ten additional commercial unrefined salts contained 95.2 ± 78.5 (2SD) ppm of Sr^62^.

As plants, and not meat, generally represent the main source of strontium in the human diet, [Sr] depend heavily on trophic level of the tested individual (i.e. herbivores will have higher [Sr] in their bones and teeth compared to carnivores), even more so than on geological variations. [Sr] therefore provides information about dietary habits rather than geographical origin^40,55,63^. Products high in [Sr] (eg. fish flour (231–280 ppm), kale (109–117 ppm), kelp (98 ppm), thyme (90 ppm), and several spices in general, clam (26 ppm)^54^) should affect the resulting Sr mix in a consumer to a larger degree than products lower in Sr, such as grains that are in the range of 1–3.8 ppm^54^, weighted according to the ingested quantity. Furthermore, Sr uptake (by plants, and thus in the rest of the food chain) is enhanced by soil acidity^64^ and Sr metabolism is highly correlated with calcium (Ca) intake, with Ca preferentially being incorporated over Sr^39,40,65^. Considering that Sr substitutes for Ca in the skeleton, high Ca levels in the diet prohibit Sr to be incorporated in the skeleton. Accordingly, high dairy consumption—besides being poor in Sr—prevents the incorporation of Sr in bone and actively reduces [Sr]^40,54,55,63^. Opposed to this, diets high in fibre and phytate available in grains, leafy vegetables and legumes promote Sr uptake and result in higher [Sr]^40,54,57^. In addition, [Sr] is also highly correlated with salinity^66^ of the food supplies. High salt intakes promote Ca excretion and eventually cause detrimental Ca degradation in the skeleton^67–69^. This allows for potential replacement by Sr, although this could not yet be observed in (short term) experimental studies^67,68^. Fenner and Wright (2014) calculated the amount of salt consumption needed to change the ^87^Sr/^86^Sr in Mayan individuals^39^, but did not account for the metabolic effects of the added salt on Ca balances in the bone. They concluded that a daily dose of 9.2 g of dietary salt per day is able to warp the ^87^Sr/^86^Sr towards sea levels in a specific diet of lime-treated (and thus Ca rich) produce, which would be considerably higher than the 5 g daily salt intake recommended by the World Health Organization^70^. Yet, this high salt intake might not be unusual, since an actual salt intake in the current world population up to even 15 g a day is not uncommon in regions such as West and East Asia^71^. It is important to keep in mind that human diets are multi-component diets meaning that Sr intakes come from a summation of different sources^65^. The final Sr signature measured in the bone thus has to be seen as a mixture of Sr resources, rather than a direct reflection of one food source.

## Results

### Radiocarbon dating

Six new dates on the identified individuals of the bone pit (identified as “gx”) (Suppl. Text 4) from the Roman period show a remarkable overlap. A chi-squared test (T = 1.472 (5% 11.071), df = 5) confirms that these individuals are contemporary and could correspond to the same chronological event between 124 and 204 cal. CE (95.4% probability). See Suppl. Text 4.

### ^87^Sr/^86^Sr in plants as bioavailable reference

Since a bioavailable Sr map for Belgium is not yet developed^72^, only preliminary results based on limited plant sample sizes are available^8,73^. While around Herstal and Fize-le-Marsal some plant data was already published^8,73^, around Destelbergen, six new sampling locations were selected (Fig. 1) within 10 km of the site. Plants were collected following the method described in Snoeck et al. 2020^74^. The ^87^Sr/^86^Sr of all 18 new plant samples (three per location) around Destelbergen result in an interquartile range (IQR) of 0.7095 to 0.7104 (Suppl. Table 4). For more information on the published local estimates of Herstal (IQR’s 0.7090–0.7099 and 0.7134–0.7141) and Fize-le-Marsal (IQR 0.7098–0.7103), see Suppl. Text 1 and^8,73^.

### ^87^Sr/^86^Sr and [Sr]

**Figure 2:**
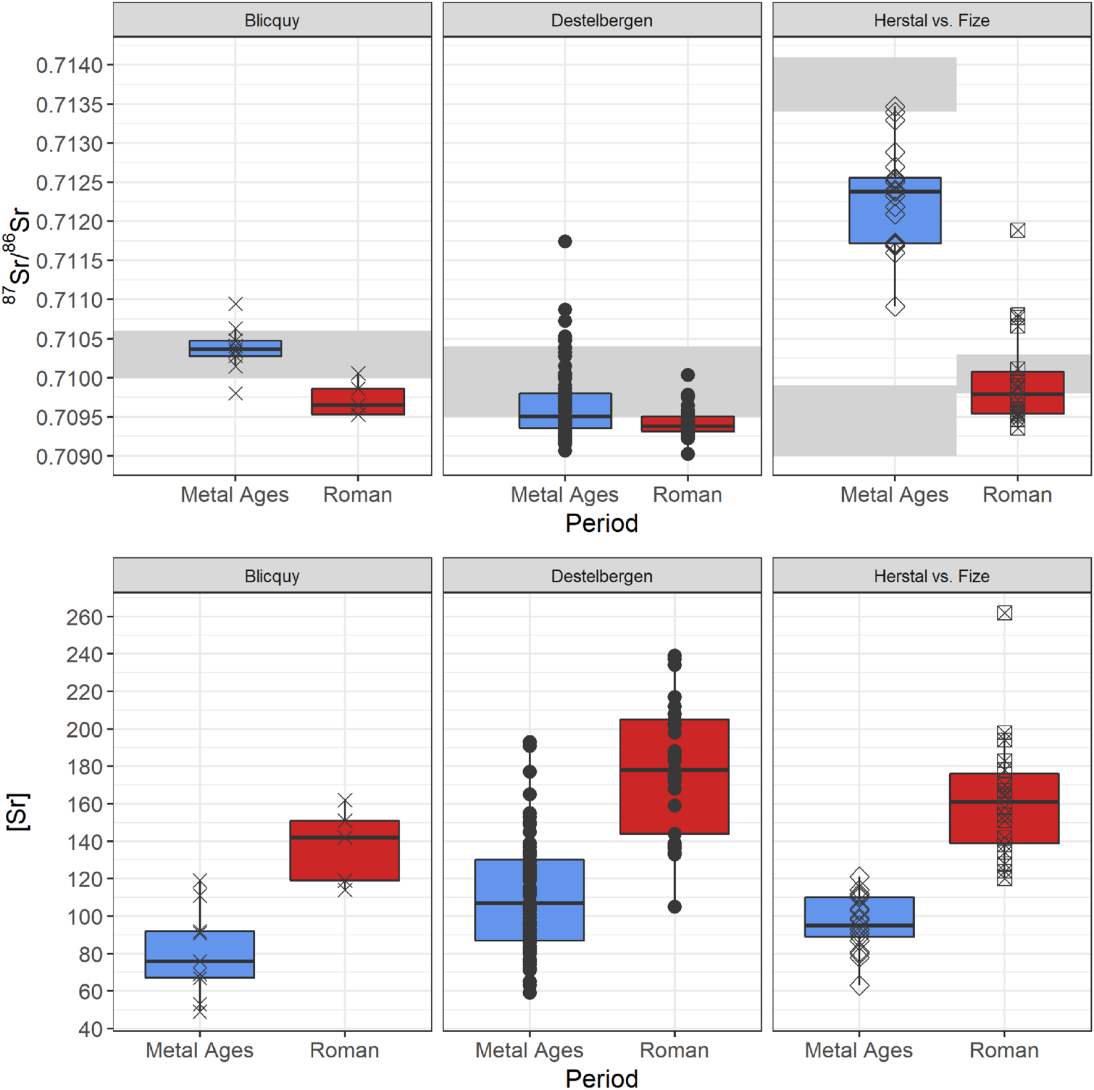
Comparison of ^87^Sr/^86^Sr and [Sr] between the Metal Ages and Gallo-Roman buried individuals of Destelbergen, Blicquy, Herstal^8^ and Fize-le-Marsal. The available ^87^Sr/^86^Sr baselines are indicated in grey. The baseline of Herstal includes two isotopically very different geological formations. [Sr] normalized to 40 wt% Ca. (graph created using R Studio (R version 4.0.2), www.r-project.org).

The ^87^Sr/^86^Sr of the Destelbergen Metal Ages group vary from 0.7091 to 0.7117 (n = 89) (Suppl. Table 2) and present a wider range (IQR 0.7094–0.7098, 0.0004) compared to the Roman group (IQR 0.7093–0.7095, 0.0002) with values ranging from 0.7090 to 0.7100 (n = 33) (Fig. 2). Although there is a great deal of overlap, a Mann–Whitney U test confirms both groups can be statistically significantly distinguished (U = 1948, *p* < 0.01). Most results fall within the IQR seen in the plant samples (0.7095–0.7104). Despite their identical baseline, the difference in ^87^Sr/^86^Sr between Metal Ages (n = 9) and Roman individuals (n = 5) in Blicquy (Suppl. Table 3b) is very clear (Mann–Whitney U test: U = 43, *p* < 0.01). The IQR of the Metal Ages individuals (0.7103–0.7105, 0.0001) does even not overlap with the IQR of the Roman individuals (0.7095–0.7099, 0.0003). Despite their relatively close proximity to each other and similar geological environment, the individuals of Herstal (published, see^8^) and Fize-le-Marsal exhibit distinct ^87^Sr/^86^Sr (Suppl. Table 3a and 3b). It has to be admitted though that in the comparison between these two sites, the baselines are not identical as is the case with the previous sites and local presence of more extreme ^87^Sr/^86^Sr are likely more at influence in Herstal. The Roman site exhibits lower results (IQR 0.7095–0.7101, 0.0005), slightly more distributed towards the lower side of the expected local bioavailable value of 0.7098, while the Metal Ages site reveals a higher range (IQR 0.7117–0.7126, 0.0008). The higher results of Herstal are not in concordance with the measured value for the local geological background (0.7092), but fall entirely in between this bioavailable range and the nearby Meuse alluvion measured further downstream (0.7136).

The Metal Ages group in Destelbergen shows four statistical outliers on 89 individuals. Both Blicquy groups show very little variability and give the impression of sites without clear mobility. Herstal on the other hand, despite its large variability, does not show statistical outliers, which suggests an absence of mobility in this group. In a region with variable bioavailable ^87^Sr/^86^Sr, however, it is difficult to distinguish non-local individuals, since many non-local signals can overlap with the locally occurring values. In the Gallo-Roman group of Destelbergen, no such outliers are present and the ^87^Sr/^86^Sr homogeneity is exceptional (IQR 0.0002). Gallo-Roman Fize-le-Marsal contains a single outlier (burial 17, 0.7119), who could be a non-local individual. Three more individuals exhibit higher ^87^Sr/^86^Sr, above 0.7106, than the majority of this group (< 0.7102).

While ^87^Sr/^86^Sr clearly shows a contraction towards seawater values in Roman times, even more contrast can be seen in [Sr]. Statistically significant differences in the [Sr] between the Metal Ages and Roman group in Destelbergen can be confirmed using the Mann–Whitney U test (U = 191.5, *p* < 0.0001), in which the Roman individuals contain higher [Sr] (IQR 144–205, 61 ppm) than their Metal Ages counterparts (IQR 87–130, 43 ppm). In Blicquy a similar shift from lower [Sr] in the Metal Ages individuals (IQR 67–92; 25) to higher [Sr] in the Roman individuals (IQR 118–160; 42) can be observed. Both sites in the Meuse valley show the same trend. Significantly higher [Sr] can be seen in the Gallo-Roman group of Fize-le-Marsal (IQR 139–176, 37 ppm), but not in the Metal Ages group of Herstal (IQR 89–110, 21 ppm). Cross-period testing of all the data with the Pairwise Wilcoxon Signed Rank test leads to a p-score < 0.0001, acknowledging them as statistically different.

Three scatterplots of the data (Suppl. Fig. 3) investigate the correlation between ^87^Sr/^86^Sr and [Sr] and clearly visualise a clustering of the Gallo-Roman individuals compared to the more dispersed Metal Ages individuals. This difference is most clear with regards to the [Sr] than for the ^87^Sr/^86^Sr taking into account the local bioavailable ^87^Sr/^86^Sr variation.

## Discussion

The ^87^Sr/^86^Sr and [Sr] obtained from cremated individuals from the Metal Ages and the Roman period in Destelbergen, especially when compared to the sites of Blicquy, Herstal and Fize-le-Marsal (Fig. 2), show that mobility, dietary practices and/or land use, considerably changed from one period to the next. As the two cemeteries in Destelbergen were found only 120 m apart and the Roman settlement was situated on top of the Metal Ages cemetery, the underlying bedrock does not cause the differences. The same can be said about Blicquy, where the same underlying bedrock did not offer different bio-available ^87^Sr/^86^Sr and [Sr] to the Metal Ages and Roman groups.

The lower variability in the ^87^Sr/^86^Sr in the Roman period in Destelbergen (and to a lesser extent in Fize-le-Marsal versus Herstal) compared to the Metal Ages groups (Fig. 2) could be explained by a reduction in mobility. However, this is in contradiction with the current archaeological and historical evidence that generally supports an increase in economic and military mobility in the Roman period compared to the Metal Ages^29,75,76^.

Changing land use strategies are able to considerably influence the Sr variability of a population. The cultivation of different areas of the landscape over time possibly exhibiting various Sr signatures, may result in the uptake of distinct ^87^Sr/^86^Sr^77^. In Destelbergen, a small sedentary group maintaining the cemetery in the Metal Ages, lived within a reasonably diverse ecosystem (dryer sand ridge, wetter fluvial plain and wetlands) supposedly allowing for some settlement continuity. Nevertheless, they likely moved the location of their settlement and crops over the course of the 650–700 year use of the cemetery^45^, varying their local Sr intake. This area in which the farmstead shifted would not necessarily have been so large, still the geological background around Destelbergen is not entirely homogeneous (Fig. 1), potentially explaining the more variable ^87^Sr/^86^Sr. The same observation applies to Herstal. Even though in Blicquy we have no locally measured baseline, the measurements of the Eocene formation in Destelbergen likely can be extrapolated to the Blicquy environment. The main occurring formations are of Ypresian (like in Destelbergen) and Thanetian age. The Thanetian immediately precedes the Ypresian, thus likely differs not much in (heavily age-dependent^52^) ^87^Sr/^86^Sr. The local baseline can be expected to be in the range of 0.7100–0.7104 (IQR Eocene formation Destelbergen). The Blicquy Metal Ages group exactly matches this expected baseline, but the Roman group falls clearly below. The much higher ^87^Sr/^86^Sr of the Herstal group than the locally underlying value of 0.7090–0.7099 (IQR), seems to be influenced by the close proximity of the Meuse alluvion (IQR 0.7134–0.7141). A mixed use of alluvion and river terrace would lead to a population with a variable ^87^Sr/^86^Sr in between both geological formations. In this site, the large diversity in ^87^Sr/^86^Sr is not in the first place connected to the timespan of the cemetery, which is with maximum 400 years shorter than in Destelbergen, but rather to the large geological diversity present locally.

The Roman settlement in Destelbergen on the other hand, located next to the Roman cemetery (on top of the Metal Ages cemetery), might indeed have exploited a more stable portion of land, since the tested sample represents the land use of a shorter period of time (mainly two centuries). Seeds and pollen confirm nearby grain fields, although the exploitation was not on an intensive scale^17^. The warmer and dryer climate in the Roman period^42^ may have led to an intensified exploitation extending into lower lying, wetter parts of the landscape, such as the fluvial plain, while during the Early Iron Age, one had to resort to the higher and drier soils. While a slightly changed and more stable agricultural land use for staple food might have been the case, there is still the established idea that in the Roman period, more consumer goods were transported than ever before. Although staple foods must have mostly been provided locally, this diversified supply nevertheless contributed to the individual’s ^87^Sr/^86^Sr and [Sr]. As such, a drop in isotopic variability contrasts expectations.

The ^87^Sr/^86^Sr in Fize-le-Marsal fits relatively well with the expected locally available ^87^Sr/^86^Sr, except for four individuals with higher results. This is not surprising, since the northwest of Belgium appears to be characterised by a large geological diversity often extending in high ^87^Sr/^86^Sr, which is reflected in the large ^87^Sr/^86^Sr ranges in the studied sites (see e.g. Herstal^8^ and Echt^73^) and sampled plants^73^. It is however apparent that the Roman groups, despite being located in regions with abundant availability of higher ^87^Sr/^86^Sr, pick up on this to a lesser extent and display often a narrower ^87^Sr/^86^Sr range than the Metal Ages groups. The ^87^Sr/^86^Sr warped towards the lower side of the baselines and the marine value of 0.7092, combined with their elevated [Sr], makes it tempting to compare the Gallo-Romans to the coastal populations of the Hebrides and Orkney^78^. The extreme marine signatures seen in these Scottish groups found on different isles (Suppl. Fig. 4) present as a relatively homogeneous, contracted ^87^Sr/^86^Sr around 0.7092 and elevated and variable [Sr], often in excess of 150 ppm. The Belgian Gallo-Roman individuals tend towards patterns shown in these marine-affected people, whereas this is not the case in the Metal Ages individuals. This marine signal in measured human samples from the Scottish Isles is evidently explained by direct salty sea spray, by Sr-rich seaweed used as a fertilizer^40,79^, and/or direct consumption of marine products^58^. Whatever the dominant source of this marine signature is, it consistently reflects the same ^87^Sr/^86^Sr of the sea and warps the other Sr resources an individual consumes at a rate depending on the amount and [Sr] of the marine resource^39^.

Since the Belgian Roman individuals tend towards a rather marine Sr signature, one should try to define the cause of this marine influence. Sea spray is not an option for the Belgian inland sites, as this effect fades out after a few kilometres^80^. Several reasons make frequent seaweed manuring in the Belgian sites unlikely. The distance from the sea meant that the recurring transportation cost to supply seaweed in large amounts were a considerable disadvantage over other types of terrestrial fertiliser. On top of that, the needed amount to alter the Sr signature of a population, once diluted through the local soil and diminished via refraction in crops, is substantial. Finally, the frequent occurrence of Gallo-Roman stables with manure accumulation pits in the sandy regions in Belgium (and even on site in Destelbergen) from the second century onwards^4,34^, prove that the use of animal manure was an established practice in the Roman period. It therefore seems unlikely that seaweed as a fertilizer would have played an important role in the available Sr budget in Destelbergen, and even less so in Fize-le-Marsal considering its location further inland. A marine-like influence could, however, have come from a change in diet with a marine component or specific landscape use.

In Destelbergen, the ^87^Sr/^86^Sr of the plant samples reveal lower values around 0.7095 in the quaternary alluvion, while on the sandy ridge adjacent to the alluvion, somewhat higher ^87^Sr/^86^Sr of 0.7102 are measured. Under an agricultural system limited to the alluvion, e.g. under the influence of the improved climate^42^, this would indeed lead to lower ^87^Sr/^86^Sr in the human individuals. Nevertheless, the settlement itself covered both geological formations and to sustain larger groups of inhabitants, one would expect that much of the surrounding landscape, including the ridge with higher ^87^Sr/^86^Sr, would be exploited. Although unexpected, it is possible that there was indeed a selective land use that could lead to lowered, contracted ^87^Sr/^86^Sr results for the Gallo-Roman group. However, this is not sufficient to explain the elevated [Sr] seen in this group.

Supplementing one’s diet with large proportions of imported food from regions with a lower ^87^Sr/^86^Sr (such as parts of the source regions of rivers Scheldt and Lys in the north of France (e.g. 0.7087^81^) could also have a lowering effect on the mixed ^87^Sr/^86^Sr. Although the possibility exists, it is highly unlikely that Destelbergen, a rural settlement which presumably lacked the means to purchase all of its grain elsewhere, would be structurally supplemented with imported staple food from other regions. Interestingly however, a seeds and pollen study of Destelbergen revealed a seed of white laceflower (*Orlaya grandiflora*), which is a Mediterranean arable weed preferring Ca-rich limy soils that are not locally present. The find of this taxon is commonly interpreted as at a certain moment being introduced via imported grain as food or seed^17,18^, hinting at potential grain transports from nearby regions such as the loamy belt in Belgium, northern France or the German Rhineland. This weed was also found in the grain cargo of a sunken Roman barge at Woerden (NL) at the Rhine limes^82^ and in Late Roman coastal castellum of Oudenburg^83^. In the context of Destelbergen, however, the singular *Orlaya grandiflora* seed likely results from an occasional introduction and not from structural grain imports, which nonetheless ties the site to a larger trade network. Importantly, structural grain imports would not necessarily explain elevated [Sr] in both Gallo-Roman rural groups.

Dietary changes are more likely to explain the elevated [Sr] seen in the Roman population than biosphere differences^40,55,63^. Increased [Sr] in itself could be caused by diets lower in dairy and/or meat^55,84^ and higher in plants^55^, but there is no direct indication that the Gallo-Romans clearly abandoned dairy and/or meat in their diet. This particular dietary change alone would also not result in a contraction to lower ^87^Sr/^86^Sr. The staple food in both Metal Ages and Roman period was based on grains and to a lesser extent on pulses^12,13^. This diet was in both periods supplemented with meat from domesticated cattle, sheep and pig. However, in the Roman period also fish products, imported foods, some newly introduced vegetables and herbs are entirely new additions. Of these, marine resources are of particular interest to explain the Sr signature shift towards more marine values in Gallo-Roman individuals.

Sea fish in itself is unlikely to transmit a marine Sr signature to human consumers very well, since Sr predominantly accumulates in the fish bone and not in soft tissue. A Sr uptake derived from fish would require the fish skeleton to be eaten as well, which is generally less probable but could be the case in fish sauce. High [Sr] measured in clams^54^ also sound promising as potential source of marine Sr, although it is unclear whether the not eaten shells were included in the measured samples. A risen sea food consumption in the Roman period, if it included fish bone, would definitely have helped in the Sr signature shift towards marine values in the Roman period, but due to the fact that the fish skeletons are usually discarded, the contribution might be not as severe as with sea salt. Due to their naturally high [Sr], salted products usually have a stronger impact on both the ^86^Sr/^86^Sr and [Sr]^64^ of human remains than untreated products. On top of that, an elevated salt consumption has an added metabolic effect. High salt diets been demonstrated to cause detrimental changes in the Ca balance in the bone^69^, while Ca reducing conditions in the skeleton are understood to lead to elevated [Sr]^40^. Salt consumption was for instance found to be at the root of clear ^87^Sr/^86^Sr shifts in Mayan samples from Tikal, demonstrating that the amount of ^87^Sr/^86^Sr warping is a function of the amount of salt consumed^39,85^. Based on the combined evidence^65^, we propose that an elevated use of (sea) salt, probably used as a food preservative, best explains the observed changes in the Sr signature from the Late Bronze Age-Early Iron Age to the Roman period in this region. The economic and social significance of salt in the Roman period, especially in connection with the maintenance of a large military force, is well-recognised and was most likely used in larger amounts than in the Metal Ages^35,37^. Long distance trade and mobile military forces must have required more edible goods to be preserved and might be a potential driver for elevated salt use. This dynamic, applied to meat, possibly developed in the Late Iron Age^31^. Additionally, a warmer Mediterranean climate that worsened the shelf life of untreated food, could have stimulated this Roman culinary trend, which in turn would influence the Northern provinces. The rise in fish consumption in the Roman period, often in salted fashion, compared to its absence in the Metal Ages, might be an important manner in which salt consumption increased. Additionally, salted hams were a famous regional and even exported product^86^, which receive much of their Sr properties from the salt used^61^. Furthermore, it must be stressed that added salt could alter, but never completely erase the Sr signature of other, dominant ingested food sources. In regions with high bioavailable ^87^Sr/^86^Sr, individuals with a diet of local produce combined with a high salt consumption would still display high ^87^Sr/^86^Sr, but somewhat lower than the bioavailable signal.

Comparing Sr signatures over time makes it possible to examine salt distribution and consumption patterns. This offers a very interesting addition to the study of salt production based on material remains from production sites. Future research should aim at exploring this shifting effect to elevated [Sr] and warped ^87^Sr/^86^Sr range on a wider scale, pinpointing when and locating where sites seem to conform to this pattern and in this way determine how trends in diet and food preservation changed.

The results of this study show a significant rise in [Sr] in Gallo-Roman Destelbergen individuals compared to their Metal Ages predecessors. This effect, also clearly observed in Blicquy and to some degree in Fize-le-Marsal, is accompanied by mildly lowered and contracted ^87^Sr/^86^Sr range towards 0.7092. These changes are best explained by an augmented salt consumption and its use as a preservative (e.g. for fish and meat). Despite the vast complexity of Sr metabolism in multi-component diets, these analyses offer an indication of connectivity (trade) and diet rather than mobility and reveal to what degree a group was tied in the wider economic and cultural fabric to obtain commodities such as salt. Circumstantial evidence is therefore needed to support such interpretations, as elemental and isotopic Sr in humans are the summation of many dietary and metabolic factors^65^. Still, this study confirms that in the absence of C, N, S isotope analyses, isotopic and elemental Sr analysis can detect dietary variations even in cremated human remains, and [Sr] can be more revealing than is often assumed. As in clearly marine populations, where locality/mobility of the individuals is often obscured due to the impact of salty sea spray and/or a marine diet, this study demonstrates that in populations with high salt use caution is required when interpreting mobility based on ^87^Sr/^86^Sr alone.

## Materials and methods

### Sampling

One hundred and twenty-two individuals (89 Metal Ages and 33 Roman) from 116 graves were sampled for ^87^Sr/^86^Sr and [Sr] analysis. Thirty-four of these sampled individuals have available ^14^C dates, of which 28 have previously been published^87^ and six new were obtained for the Roman bone pit (gx) with multiple individuals (Suppl. Text 1). Unfortunately, only a limited number of Roman individual graves could be sampled and of these all bone samples were too small for ^14^C dating. The same bone fragments used for ^14^C dating were used for ^87^Sr/^86^Sr and [Sr] analyses. In graves that were not ^14^C dated, calcined diaphysis fragments were selected when available. The fragmentary state of 23 individual graves made sampling of diaphyseal fragments impossible resulting in the selection of two cranial fragments and 21 unidentifiable human bone fragments instead. In the bone pit (gx), seven right parts of mandible were selected (six of which were radiocarbon dated) as these skeletal elements can be used to infer the grave’s minimum number of individuals (MNI). Two of these individuals were identified to be nonadults. Four extra samples from the bone pit were analysed (juvenile rib, adult rib, juvenile diaphysis and adult diaphysis), but as these samples cannot conclusively be identified as separate individuals, these extra data were not included in the statistics and are merely added in Suppl. Table 2.

Nine Metal Ages graves of Blicquy were analysed (eight diaphyseal and one cranial fragment). Five of Blicquy’s Roman graves (diaphysis fragments) were analysed and also ^14^C dated.

Even though the results of several samples per grave of Herstal were available to study life biographies, bringing to light that in some graves the remains of several individuals were present^8^, for this study only one sample per grave was (randomly) selected. This ensures that every individual is only accounted for once. This way, Herstal delivered twenty-one samples (all diaphysis fragments) for this study. The selection of these previously published results is listed in Suppl. Table 3a.

Eighteen cremated fragments from Fize-le-Marsal were analysed. All were diaphysis fragments, except for grave 14, for which a cranial fragment was selected in the absence of diaphyseal remains. For reference, four Sr samples from Fize-le-Marsal have been radiocarbon dated (Suppl. Table 3b).

The cremated remains under study are owned by museal and research institutions. The owners have confirmed that limited destructive analysis was allowed for the purpose of this study. Ethical and academic guidelines concerning the study of archaeological human remains set by the Flemish government have been followed during the research. Further administrative and ethical information regarding the cremated remains of these sites can be consulted in Suppl. Text 5.

### Sr isotope and concentration analysis

Snoeck et al. (2015) demonstrated that Sr isotope analysis is possible on calcined bone, which holds, thanks to its high crystallinity, even less contamination than previously favoured tooth enamel^5^. The pretreatment, extraction and mass spectrometry measurements of the samples were performed at the AMGC laboratories at the Vrije Universiteit Brussel (VUB) and G-Time laboratories at the Université Libre de Bruxelles (ULB). The procedures described in Snoeck et al. 2015^5^ were applied. The samples were first mechanically cleaned by drilling off the possibly contaminated outer layer. Next, chemical cleaning was done by three series of 10 min of ultrasonication in milliQ water, followed by one time of 3–10 min of ultrasonication in 1 M acetic acid. Finally, another three times of ultrasonication in milliQ water finishes the cleaning process. The cleaned samples are then dried and powdered. The extraction process is described in Snoeck et al. (2015)^5^. Columns filled with Sr-specific resin (Eichrom Sr Spec) separated the Sr from the sample. The calculated Sr column recovery rate yields more than 95% of total Sr. As a reference for testing the accuracy of the subsequent analytical measurements, a sample of the standard ‘bone ash SRM1400’ underwent the same extraction process.

Most measurements of the ^87^Sr/^86^Sr were executed on a Nu Plasma MC-ICP Mass Spectrometer (Nu015 from Nu Instruments, Wrexham, UK) at ULB while samples 07209 to 07231 were measured at the VUB on a Nu Plasma 3 (PD017 from Nu Instruments, Wrexham, UK). Repeated measurements of the NBS987 and SRM1400 standards yielded ^87^Sr/^86^Sr = 0.710246 ± 45 (2SD for > 300 analyses) and 0.713159 ± 30 (2SD; n = 21) respectively. For this research purpose, this is sufficiently consistent with the mean value of 0.710252 ± 13 (2SD for analyses) obtained by Thermal Ionization Mass Spectrometry (TIMS)^88^. A standard bracketing method with the recommended value of ^87^Sr/^86^Sr = 0.710248 was used to normalise all sample measurements^88^. Procedural blanks were considered negligible (total Sr (V) of max 0.02 versus 7–10 V for analyses, equivalent to ≈ 0.3%). The ^87^Sr/^86^Sr is reported with a 2SE for each sample (absolute error of the individual sample analysis—internal error).

An aliquot of 0.5 ml of all dissolved samples was used to measure the Sr and Ca concentrations. Once diluted again with 0.42 M HNO_3_, a Thermo Scientific Element 2 sector field ICP mass spectrometer at Vrije Universiteit Brussel (VUB) was used to determine the Sr and Ca concentrations in low and medium resolution respectively using indium (In) as an internal standard and external calibration versus various certified reference materials (SRM1400, CCB01). The strontium data were then normalised to 40 wt% Ca to account for the varying loss of organic matter and carbonates during cremation. All [Sr] mentioned in the text and supplementary data refer to Sr concentrations normalised to 40 wt% Ca. To evaluate the accuracy of the procedure, two internal bioapatite standards (ENF and CBA) were analysed in parallel. Based on repeated digestion and measurement of these reference materials, the analytical precision of the procedure is estimated to be better than 5% relative standard deviation (1SD, n = 33 for CBA and n = 5 for ENF). For the procedures used to prepare the plant samples and produce the Sr baseline map, see Veselka et al. 2021^73^ and Snoeck et al. 2020^74^.

### ^14^C dating

The ^14^C dating of the bioapatite in the cremated bone samples, six of Destelbergen, four of Fize-le-Marsal and five for Blicquy, was performed at the Royal Institute for Cultural Heritage (KIK-IRPA, Brussels, Belgium) and followed the KIK-IRPA protocol^89^. Radiocarbon dates were calibrated using the software OxCal 4.4^90,91^ and the IntCal20 calibration curve^92^.

### Data processing

Data management, plots and statistical tests were performed with R Studio (R version 4.0.2 (2020–06-22). Maps were compiled with QGIS version 3.12.0-București. Data are openly available in the IsoArcH database https://www.isoarch.eu^93^.

## Supplementary Information

Supplementary Information.

## Data Availability

All data are available in the main text or the supplementary materials.
