# Supplementary material for: Strontium isotopes and concentrations in cremated bones suggest an increased salt consumption in Gallo-Roman diet
Source: Sci Rep. 2022 Jun 3;12:9280. doi: 10.1038/s41598-022-12880-4 (PMC9166795; doi:10.1038/s41598-022-12880-4)
Supplement: Supplementary file 1 — Supplementary Information. [file 41598_2022_12880_MOESM1_ESM.pdf]

Supplementary information for

Strontium isotopes and concentrations in cremated bones suggest an increased salt consumption in Gallo-Roman diet

Sarah Dalle*, Christophe Snoeck, Amanda Sengeløv, Kevin Salesse, Marta Hlad, Rica Annaert, Tom Boonants, Mathieu Boudin, Giacomo Capuzzo, Carina T. Gerritzen, Steven Goderis, Charlotte Sabaux, Elisavet Stamataki, Martine Vercauteren, Barbara Veselka, Eugène Warmenbol, Guy De Mulder

*Corresponding author. Email: Sarah.Dalle@ugent.be

Supplementary Text 1

Destelbergen is a complex site consisting of two main areas (Fig. S1). The northern part revealed a substantial Late Bronze Age to Early Iron Age cemetery (1100 – 450/400 BCE) of about 7.000 m² that was later partly overbuilt by a dense Gallo-Roman (52 BCE – 406 CE) settlement. Less than 150 m to the south, a large Gallo-Roman cemetery of more than 22.800 m² was discovered. All excavated graves were cremation burials, as this was the dominant custom in both periods for Belgium[1]. In the Metal Ages cemetery (107 burials), a general chronological evolution from east to west was found[2]. In the Gallo-Roman cemetery, which was with its 204 burials far more extensive than most common familial cemeteries of this period[3], graves were usually simple pits. One notable exception to these uniform burials is a unique so-called 'collective grave' (gx), which is very rare in this area. The atypically large pit of 13.3 m long contained 17.5 kg of bone and well over 200 kg of grave goods associated with these cremation deposits. Based on the find of 12 right petrous bones, the minimum number of individuals (MNI) is 12[4], but a larger number of people is likely when taking into account the bone weight and amount of grave goods.

No traces of a Metal Ages settlement were found. In the Gallo-Roman settlement, the organisation, density and variety of material finds and the extent of the accompanying cemetery indicate that a modest settlement developed into a more organised, larger, and wealthier centre than the average farmsteads common to the region, but not as elaborate and complex as a classic *vicus*[5]. It is possible that the site accommodated a minor administrative organisational presence, possibly offering a place for trade and crafts such as metal and wood working[5]. The place was undoubtedly a well-connected local hub in the societal web in the context of the Scheldt river. The river functioned as a transport corridor between northern Gaul, the Rhine *limes* and Britain, which is evidenced by the presence of several ports and quays along its course[6] (see Text S3). The lack of military evidence suggests, however, that the Destelbergen settlement itself was civilian in nature.

A location at the Scheldt banks meant an easy connection to the North Sea, although before 800-1200 CE the Oosterschelde further north was then its estuary, instead of the current Westerschelde[7,8]. The route to the sea via river (most suitable for cargo transport) was therefore relatively long compared to the distance from the sea. The route via river would have been about 133 km long, calculated by GIS, with an approximation of the then present meanders before canalisation, based on soil and historical maps. This corresponds with roughly 2 days at 65km/day downstream speed, put forward by ORBIS, The Stanford Geospatial Network Model of the Roman World[9]. Even though marine influence in the coastal area increased in the last centuries BCE[7,10], Destelbergen was during the Metal Ages and Roman period never closer than 45 km from the open sea and 35 km from the dynamic coastal marshlands (current coastal plain) in which tidal inlets had free rein. General travel on foot to the coast would have taken at least a day. For average, civilian travel on foot, with or without pack animals, ORBIS estimates

30km per day as a feasible average. Ox carts are a lot slower (12km per day), while private vehicular transport can be faster (36km per day)[9] depending on the state of the roads. The population in Destelbergen can therefore not be regarded as a coastal community in either of the studied periods.

Herstal and Fize-le-Marsal, situated in relatively close proximity to each other (18 km), further inland in the Meuse basin, display an overlap with Destelbergen in terms of chronology and type of site. Furthermore, their similar local bio-available Sr isotopic signature between themselves despite being located in different lithologies makes them interesting to compare. The urnfield of Herstal "Pré Wigier"[11,12], like Destelbergen overlooking the river alluvion, spans the Late Bronze Age (second half of the 12th to the beginning of the 8th century BCE), tending to the earlier burials of Destelbergen, with two burials even extending to potentially 1200 BCE (2σ). The 21 graves were all simple pits, except one (grave 2), which was possibly covered by a mound. Cremated bones were usually deposited in urns or in perishable containers. Several graves proved to contain more than one individual. For the detailed bioarchaeological study, see Sabaux et al. (2021)[12]. The Gallo-Roman cemetery of Fize-le-Marsal "Beauflot-Pivache"[13] consisted of 25 burials and was found in proximity of rural settlement features. All graves were simple pits containing cremated bones either scattered in the pit, or deposited in clusters, but never held together in an urn, although most burials did include some grave goods such as pottery. One grave was recognised as a *bustum* type. The site was, like Destelbergen, dated from the first century to the beginning of the third century CE and must have benefited from its location close to a Roman road connecting the municipium of Tongeren and the *vicus* of Arlon, in a densely populated and fertile area in the Roman Period[13].
The site of Blicquy "Ville d'Anderlecht" was excavated in many phases mainly between the 1980s and 2003[14]. The location revealed under Roman building foundations 35 Metal Ages graves dating from the end of the MBA until the EIA[15,16] of which 10 could be analysed in this paper. The graves of this urnfield have recently been analysed for carbon and oxygen isotopes to reconstruct the cremation characteristics[16]. In the LIA this location was transformed into a ritual zone with offering pits[17]. In the Roman period, an extensive Roman walled sanctuary with adjoining infrastructure was installed.[17] The Roman cemetery of Blicquy "Champ de la Chaussée Trouée" belonged to the *vicus* Blicquy at the location "Camp Romain"[18]. This *vicus* is situated on the road connecting the *civitas* of Bavay with on the one hand the Ghent region and on the other the North Sea. The cemetery, at 2500 metres to the east of the Metal Ages cemetery and 275 metres southwest of the *vicus*, was excavated in the 1960s and existed of 407 graves dating from the first to the third century CE[19]. Cremated bone samples from five of these graves have been analysed in this paper.
A final Sr isotope map for Belgium is still in the making, but according to first results (see preliminary Sr map in Veselka et al. 2021[20]), both sites of Herstal and Fize-le-Marsal are situated in geological formations with comparable $^{87}Sr/^{86}Sr$ (Herstal: unit of Houiller (Carboniferous); Fize-le-Marsal: unit of Jauche (Cretaceous)). For Herstal, based on published plant samples taken close by the site, the local $^{87}Sr/^{86}Sr$ is around 0.7090-0.7099 (interquartile range (IQR) of sample locations L01-b and L02-b, each delivering three samples) and the Meuse alluvion was measured 0.7134-0.7141 (IQR sample location BV02 with four samples) further downstream[12,20]. For a complete overview of the Sr results of the human bones from Herstal, see Sabaux et al. (2021)[12]. Fize-le-Marsal likely exhibits a $^{87}Sr/^{86}Sr$ of 0.7098-0.7103 (IQR published sample location BV06 with three samples), based on a similar geological setting sampled near the site of Echt[12,20]. Generally speaking, this region around the Meuse has proven to be very diverse in lithology and local $^{87}Sr/^{86}Sr$, leading to populations with varied $^{87}Sr/^{86}Sr$ results as a consequence[20]. Both Metal Ages and Roman cemeteries of Blicquy are located on a Thanetian formation (unit of Hanut; Paleocene), with in close proximity the Ypresian unit of Kortrijk (Eocene) and smaller pockets of Holocene alluvion. The same biosphere surrounded the buried Metal Ages and Roman individuals during life offering a valuable comparison. Unfortunately, no sampling to reconstruct a baseline has taken place yet.

Supplementary Text 2.

De Mulder (2011) conservatively estimated that the Destelbergen Metal Ages cemetery would be maintained by an average population of no more than 7 to 11 people at a time (a population the size of one nuclear family)[21]. This cautiously calculated number is lower than expected for a community of several families living in the area sharing a communal burial ground as the wandering farmsteads model suggests[21]. The Acsádi and Nemeskéri calculation[22] was used and took into account known and expected destroyed graves[21]. According to osteological analyses, all age categories were represented in the Destelbergen cemetery[23].

Supplementary Text 3.

To confirm that the concentration of strontium ([Sr]) present in bone apatite remains unaltered during heating (i.e. the cremation process), [Sr] and calcium concentrations ([Ca]) of 65 cow bone fragments obtained from the same cow tibia were measured using sector field ICP-MS (ATTOM ES from Nu Instruments, Wrexham, UK). These fragments were burned for different durations (0.5 to 24h) and at different temperatures (500 to 900°C; for more details, see [24]). The [Ca] vary between 33 and 52 wt% (40.6 ± 3.5 wt%, 1 SD), which is linked to the analytical uncertainty, the uncertainty linked to the sample manipulation (including weighing and pipetting) and the variable amounts of carbonates and organic matter present in bone burned for different time intervals and at different temperatures. To account for this, the [Sr] are normalised to 40 wt% Ca, the expected [Ca] in bone apatite based on stoichiometry. The results (Suppl. Table 1 and Suppl. Figure 2) clearly show that there is no difference in [Sr] between bone fragments burned for different durations and at different temperatures, confirming that Ca-normalised strontium concentrations ([Sr]*) can be used as a reliable proxy in cremated bone.

Supplementary Text 4.

The six dated identified individuals of the Gallo-Roman bone pit (gx) belong to the second half of the second century CE and appear contemporary. This is slightly earlier than the previously postulated closing date of 200-225 CE based on the associated grave goods but does agree with the majority of the grave goods[25].

These new radiocarbon dates deliver a strong indication for the extensive bone pit with many individuals to be a one-time event, but this interpretation must still be considered with caution, seeing the presence of (less abundant) older, Flavian pottery and younger, up to early third century material in the pit. It cannot be excluded that the bone pit was an accumulation of single burials of different date with a very strong dominance of burials from the 150 – 200 CE period. It has to be mentioned that a thorough reorganization and rebuilding of the settlement has been dated exactly to that period[5]. These two determinations could equally pinpoint a period of rapid growth leading to a necessary relocation of certain graves, and/or of sudden catastrophe (such as a pandemic, raiding or other crises) in the settlement, or even a combination of both. Both types of events could cause a need to deposit remains collectively in a charnel or ossuary pit or in a communal grave.

Supplementary Text 5.

Destelbergen and Blicquy (Roman part)

The human remains from Destelbergen were excavated during the years 1960-2000 before guidelines and regulations were in place. Nevertheless, the excavations took place in a careful and respectful professional academic setting. The excavation methods in place at that time formed the basis of the current code of conduct[26]. In that respect, the current regulations were applied during excavation. To this day, the human remains remained in storage with the original excavating institution and owner, Ghent University. No cultural or ancestral claims on these human remains have been disclosed. The current study project loaned the cremated remains directly from the owner.

Contact information:
Ghent University Museum. Contact person for the archaeological remains: dr. Patrick Monsieur, Krijgslaan 281, building S30, 9000 Gent, Belgium
Currently, the samples under study in this paper have been re-inventoried only in part subsequent to the creation of the new Ghent University Museum in 2020. These 'AMUG_' codes have been added to the Grave names in Suppl. Table 2. The other remains were not yet assigned a new inventory number and are identified by their site name 'Destelbergen Eenbeekeinde' and burial number assigned during initial research and publication[4,27,28]. The Blicquy samples are identified by their original grave numbers.

Fize-le-Marsal and Blicquy (Metal Ages part)
The cremated remains of Fize-le-Marsal are owned by the Musée Communal d'Archéologie Hesbignonne, and those of Blicquy by Archéosite et Musée d'Aubechies-Beloeil asbl. The grave numbers mentioned in this article refer to the reference numbers used by the respective museums and published by the researchers[13,14].

Ethical and academic guidelines
This study followed academic and ethical guidelines set by the Flemish government[26] within Belgium. Permission for limited destructive analyses (eg. ^{14}C dating, $^{87}Sr/^{86}Sr$ analysis) on fragments of bone was granted by the respective owners. Storing and packaging regulations[29] are strictly followed.

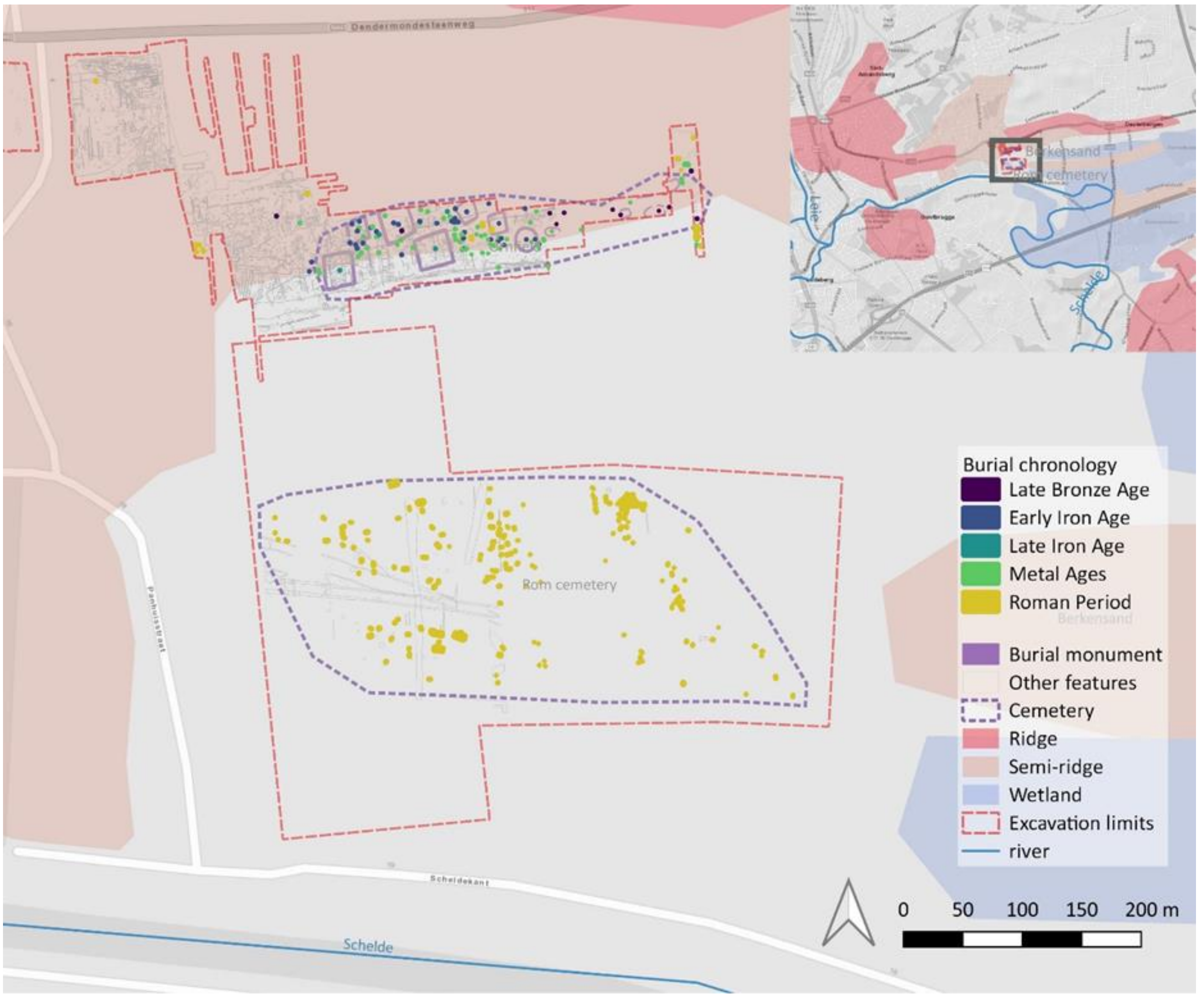

Supplementary Fig. 1.

Overview map of the excavated Metal Ages and Roman cemeteries in Destelbergen (data see [5,27,28,30,31]; map created using QGIS version 3.12.0-București, https://qgis.org/).

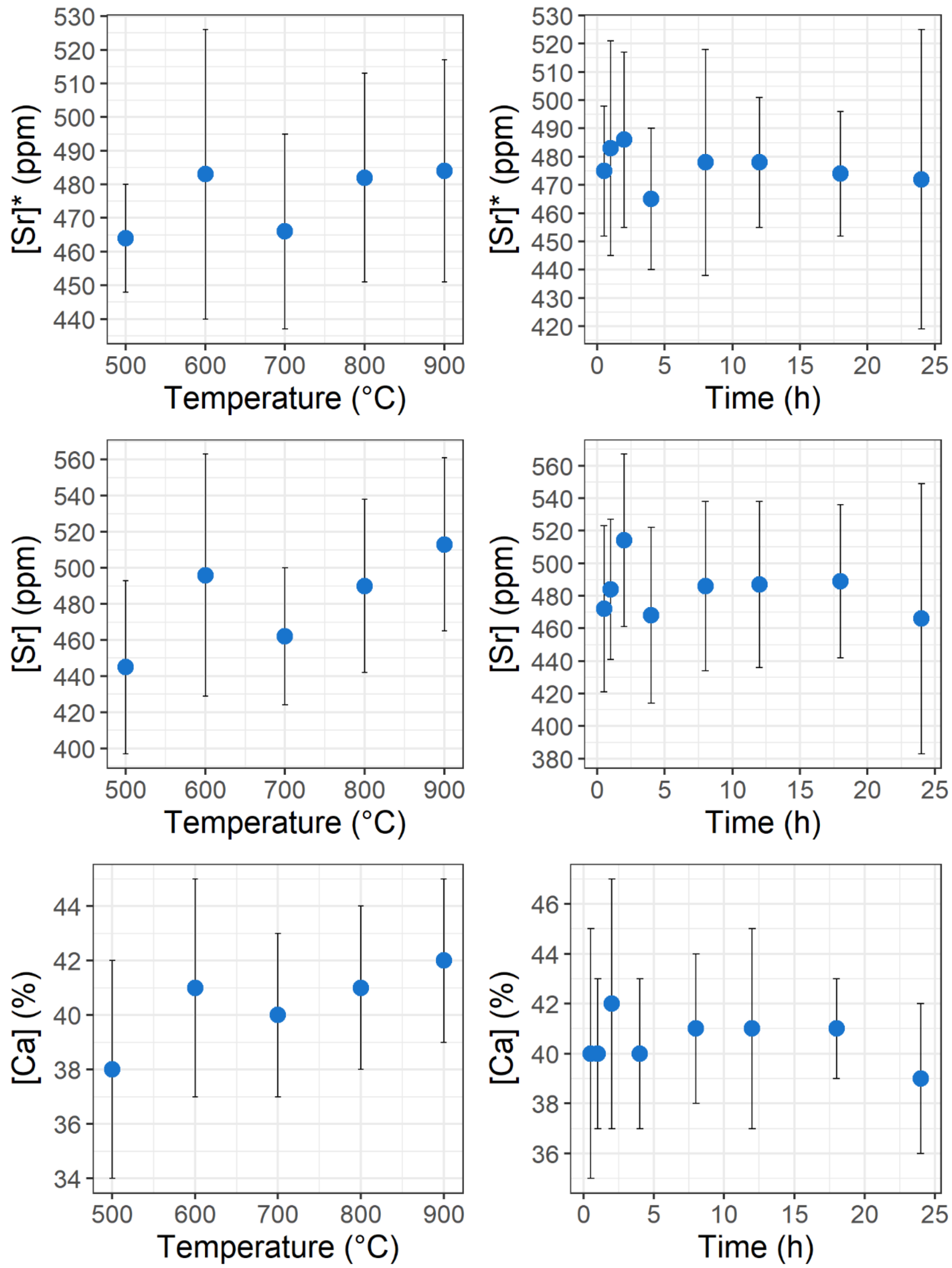

Supplementary Fig. 2.
[Sr]* (ppm) (normalized to 40 wt% Ca), [Sr] (ppm) and [Ca] (%) of cattle bone burned at different temperatures and for different durations (graph created using R Studio (R version 4.0.2), www.r-project.org).

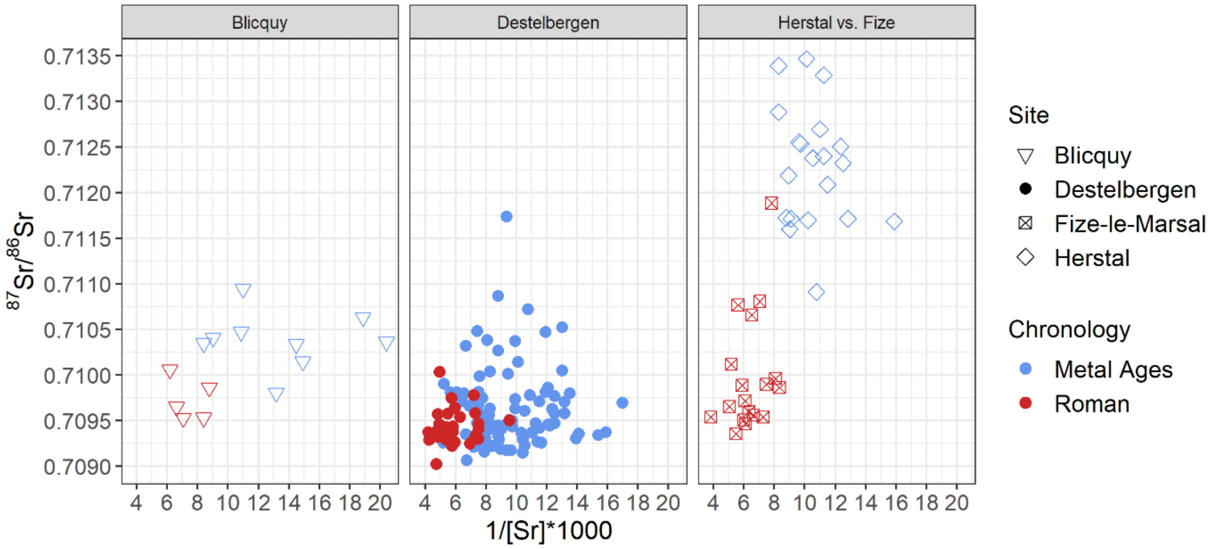

Supplementary Fig. 3.
Scatterplot of the Sr results combining 1/[Sr]*1000 and $^{87}Sr/^{86}Sr$ of Destelbergen, Herstal (data from Sabaux et al. (2021) [12]) and Fize-le-Marsal (graph created using R Studio (R version 4.0.2), www.r-project.org).

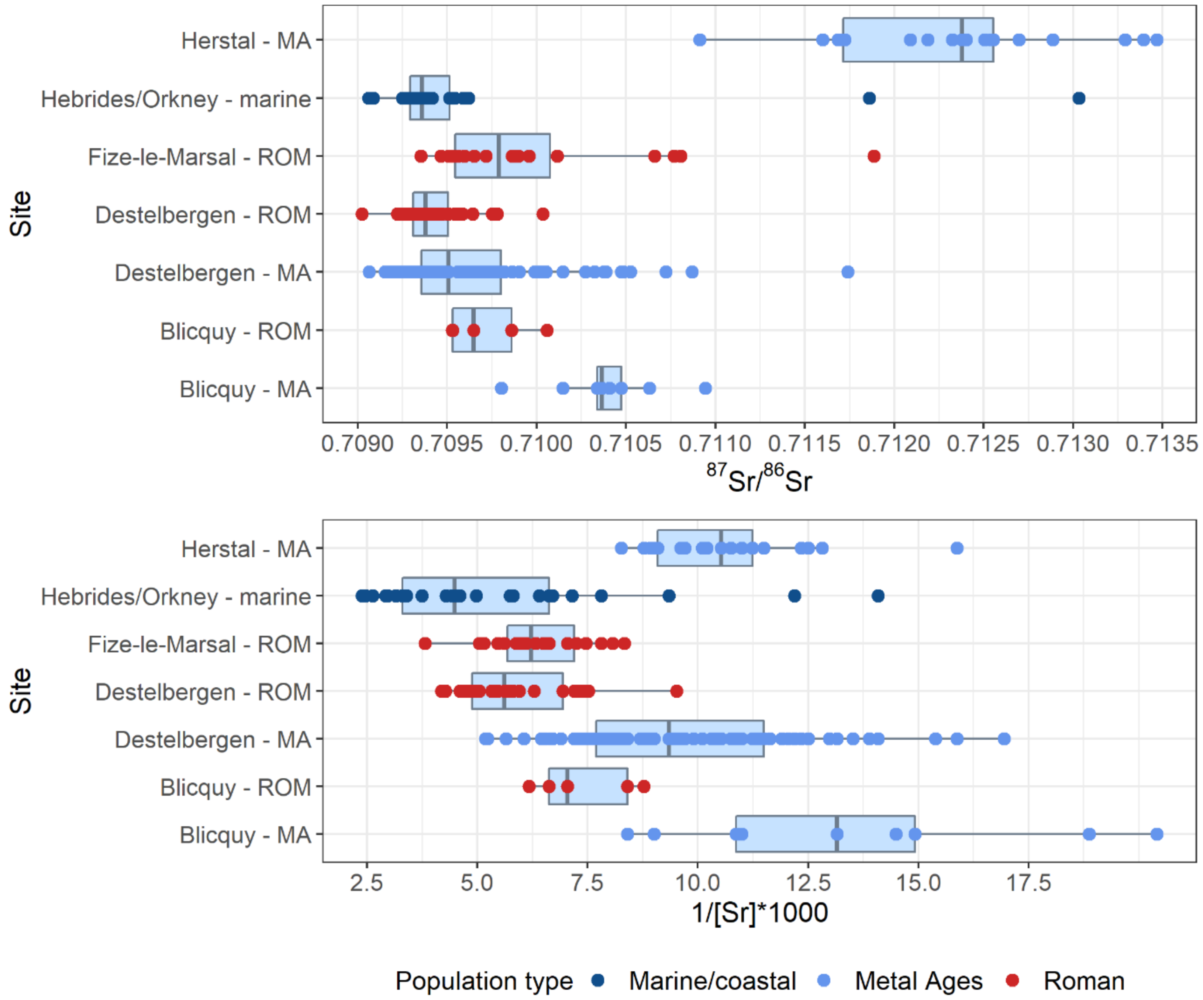

Supplementary Fig. 4.
Comparison of the Metal Ages and Roman populations of Belgium with the coastal populations with marine signal of the Hebrides and Orkney (British data derived from Evans et al. 2012[32]; graph created using R Studio (R version 4.0.2), www.r-project.org).

Supplementary Table 1a.
Average [Sr]* (ppm) (normalized to 40 wt% Ca), [Sr] (ppm) and [Ca] (%) measured on 65 cow bone fragments from the same cow tibia[24] burned at different temperatures (500 to 900°C) and for different durations (between 0.5 and 24h). Note that the RSD is slightly higher than the accepted RSD of 5%. This can, however, be explained by the variability of [Sr] within a bone.

**Temperature (°C)**	**500**	**600**	**700**	**800**	**900**	**ALL**
n	10	12	12	16	15	65
Average [Sr]* (ppm)	464	483	466	482	484	477
1SD	16	43	29	31	33	32
RSD	3.4%	9.0%	6.2%	6.4%	6.8%	6.7%

**Temperature (°C)**	**500**	**600**	**700**	**800**	**900**	**ALL**
n	10	12	12	16	15	65
Average [Sr] (ppm)	445	496	462	490	513	484
1SD	48	67	38	48	48	54
RSD	10.9%	13.5%	8.3%	9.8%	9.4%	11.2%

**Temperature (°C)**	**500**	**600**	**700**	**800**	**900**	**ALL**
n	10	12	12	16	15	65
Average [Ca] (%)	38	41	40	41	42	41
1SD	4	4	3	3	3	4
RSD	9.3%	10.2%	6.4%	8.0%	7.4%	8.7%

**Time (h)**	**1**	**1**	**2**	**4**	**8**	**12**	**18**	**24**
n	7	9	10	8	6	9	8	8
Average [Sr]* (ppm)	475	483	486	465	478	478	474	472
1SD	23	38	31	25	40	23	22	53
RSD	4.9%	7.9%	6.4%	5.5%	8.4%	4.7%	4.6%	11.3%

**Time (h)**	**1**	**1**	**2**	**4**	**8**	**12**	**18**	**24**
n	7	9	10	8	6	9	8	8
Average [Sr] (ppm)	472	484	514	468	486	487	489	466
1SD	51	43	53	54	52	51	47	83
RSD	10.9%	8.9%	10.4%	11.5%	10.7%	10.4%	9.7%	17.7%

**Time (h)**	**1**	**1**	**2**	**4**	**8**	**12**	**18**	**24**
n	7	9	10	8	6	9	8	8
Average [Ca] (%)	40	40	42	40	41	41	41	39
1SD	5	3	5	3	3	4	2	3
RSD	11.3%	7.0%	11.6%	7.7%	7.4%	9.3%	5.6%	7.9%

Supplementary Table 1b.
[Ca] (%), [Sr] (ppm) and [Sr]* (ppm) (normalized to 40 wt% Ca) measured on 65 cow bone fragments from the same cow tibia[24] burned at different temperatures (500 to 900°C) and for different durations (between 0.5 and 24h).

Time (h)	Temperature (°C)	[Ca] (%)	[Sr] (ppm)	[Sr]* (ppm)
1	500	42	494	467
1	500	37	433	466
2	500	37	446	478
2	500	46	546	476
4	500	38	422	442
4	500	36	402	449
12	500	33	378	453
15	500	36	446	492
18	500	37	415	449
18	500	40	469	473
0.5	600	43	558	517
1	600	40	540	536
2	600	52	587	455
2	600	38	486	517
4	600	38	421	446
4	600	42	550	522
12	600	43	504	466
12	600	41	469	463
18	600	42	522	499
18	600	42	486	468
24	600	35	338	386
24	600	38	488	520
0.5	700	37	448	482
0.5	700	37	436	468
1	700	37	416	445
1	700	39	525	543
2	700	41	447	439
2	700	41	470	456
4	700	42	478	458
8	700	39	421	435
8	700	37	437	468
12	700	45	526	464
24	700	39	436	453
24	700	42	502	483
0.5	800	39	458	469
0.5	800	35	418	479
1	800	42	467	444
1	800	37	474	514
2	800	40	509	512
2	800	40	512	507
4	800	40	470	474

4	800	46	537	469
8	800	46	512	449
8	800	39	531	538
12	800	41	509	498
12	800	44	534	486
18	800	43	523	484
18	800	45	567	509
24	800	37	422	459
24	800	38	398	417
0.5	900	38	455	473
0.5	900	48	530	439
1	900	41	472	462
1	900	45	532	471
2	900	40	536	535
2	900	50	598	482
4	900	40	464	462
8	900	40	466	462
8	900	43	549	516
12	900	42	482	459
12	900	41	534	521
18	900	41	479	466
18	900	40	450	448
24	900	42	564	538
24	900	44	579	521

Supplementary Table 2.

Overview of ^{14}C, $^{87}Sr/^{86}Sr$ and [Sr] of Destelbergen (*^{14}C published in De Mulder et al. 2009[33], performed on cremated bone, unless otherwise stated; **[Sr] normalised to 40 wt% Ca).

Sample ID	Grave number	^{14}C: Lab-code (Age BP ± σ)	Chronology	Bone type	$^{87}Sr/^{86}Sr$	2σ	[Sr] (ppm)**
7000	4	KIA-34909 (2120±30)*	Late Iron Age	Diaphysis	0.709903	0.000011	191
7001	6	NA	Metal Ages	Diaphysis	0.709409	0.000009	111
7002	7	NA	Metal Ages	Diaphysis	0.709449	0.000010	120
7003	8	NA	Early Iron Age	Diaphysis	0.709508	0.000012	105
7004	9	KIA-34178 (2360±30)*	Early Iron Age	Diaphysis	0.709487	0.000014	122
7005	10	KIA-34923 (2775±30)*	Early Iron Age	Diaphysis	0.710042	0.000013	121
7006	11	NA	Metal Ages	Diaphysis	0.709819	0.000021	133
7007	12	KIA-34892 (2495±30)*	Early Iron Age	Diaphysis	0.709799	0.000015	74
7008	14	NA	Metal Ages	Diaphysis	0.709816	0.000014	84
7009	15	NA	Early Iron Age	Diaphysis	0.709740	0.000018	101
7010	17	NA	Early Iron Age	Diaphysis	0.709715	0.000010	155
7011	18	KIA-34910 (2330±35)*	Late Iron Age	Diaphysis	0.709715	0.000010	87
7012	19	NA	Early Iron Age	Diaphysis	0.710051	0.000010	77
7013	20	NA	Early Iron Age	Diaphysis	0.709750	0.000015	137
6125	21	NA	Metal Ages	Diaphysis	0.709401	0.000011	123
7014	22	KIA-37706 (2405±40)*	Early Iron Age	Diaphysis	0.709302	0.000012	96
6126	23	NA	Metal Ages	Diaphysis	0.709191	0.000008	112
7015	24	NA	Metal Ages	Diaphysis	0.709212	0.000012	139
7016	25	NA	Metal Ages	Diaphysis	0.709411	0.000016	115
7017	26	KIA-35363 (2590±30)*	Early Iron Age	Diaphysis	0.709473	0.000011	135
7018	27	NA	Early Iron Age	Diaphysis	0.709862	0.000010	83
7019	28	NA	Metal Ages	Diaphysis	0.709750	0.000011	129
7020	29	NA	Early Iron Age	Diaphysis	0.709674	0.000009	139
7021	30	NA	Metal Ages	Diaphysis	0.709162	0.000009	127
7022	31	NA	Metal Ages	Diaphysis	0.710377	0.000009	101
7023	32	KIA-35353 (2370±30)*	Early Iron Age	Diaphysis	0.710387	0.000010	124
7024	35	KIA-comb (2500±60)*	Early Iron Age	Diaphysis	0.709501	0.000010	103
7025	36	KIA-30041 (2480±30)*	Early Iron Age	Diaphysis	0.710013	0.000009	106
7026	37	NA	Metal Ages	Diaphysis	0.710324	0.000009	150
7027	38	NA	Metal Ages	Diaphysis	0.709445	0.000012	82
7028	39	NA	Metal Ages	Diaphysis	0.709369	0.000009	91
7029	40	NA	Metal Ages	Diaphysis	0.709343	0.000016	65
7030	41	KIA-37707 (2450±30)*	Metal Ages	Diaphysis	0.709606	0.000014	95
7031	42	NA	Metal Ages	Diaphysis	0.709260	0.000009	130
7032	43	NA	Early Iron Age	Diaphysis	0.709357	0.000015	71
7033	44	NA	Metal Ages	Diaphysis	0.709636	0.000009	101
7034	45	NA	Metal Ages	Diaphysis	0.709628	0.000009	81
7035	46	NA	Early Iron Age	Diaphysis	0.709352	0.000014	135
7036	47	NA	Early Iron Age	Diaphysis	0.709311	0.000008	126
7037	48	NA	Early Iron Age	Diaphysis	0.709351	0.000009	150
7038	49	NA	Metal Ages	Diaphysis	0.709304	0.000014	72

7043	52	KIA-40553 (2755±30)*	Late Bronze Age	Diaphysis	0.709261	0.000007	193
7044	53	NA	Metal Ages	Diaphysis	0.709583	0.000012	76
6127	54	NA	Late Bronze Age	Diaphysis	0.709370	0.000014	97
6128	56	NA	Metal Ages	Diaphysis	0.709460	0.000013	82
7045	57	KIA-30042 (2215±30)*	Late Iron Age	Diaphysis	0.709707	0.000014	76
7046	58	NA	Metal Ages	Diaphysis	0.710272	0.000007	114
6129	59	NA	Early Iron Age	Diaphysis	0.709659	0.000013	145
7047	61	NA	Metal Ages	Diaphysis	0.709268	0.000010	88
6130	62	NA	Metal Ages	Diaphysis	0.709801	0.000016	153
7048	63	NA	Early Iron Age	Diaphysis	0.709221	0.000009	119
6131	64	NA	Late Bronze Age	Diaphysis	0.710473	0.000013	84
7049	65	KIA-35364 (2515±30)*	Early Iron Age	Diaphysis	0.709339	0.000009	114
7050	66	NA	Metal Ages	Diaphysis	0.709375	0.000011	63
-	68	KIA-36925 (2810±25)*	Late Bronze Age	NA	NA	NA	NA
7051	69	KIA-37582 (2820±30)*	Late Bronze Age	Diaphysis	0.709437	0.000014	113
6132	70	NA	Metal Ages	Diaphysis	0.709438	0.000008	103
7052	71	KIA-34887 (2320±30)*	Late Iron Age	Diaphysis	0.709699	0.000012	59
7053	72	NA	Metal Ages	Diaphysis	0.709772	0.000008	80
7054	73	NA	Metal Ages	Diaphysis	0.709471	0.000011	80
7055	74	NA	Early Iron Age	Diaphysis	0.710724	0.000012	93
7056	75	KIA-34922 (2420±30)*	Early Iron Age	Diaphysis	0.709674	0.000009	133
7057	76	NA	Metal Ages	Diaphysis	0.709227	0.000012	95
7058	77	NA	Metal Ages	Diaphysis	0.709256	0.000011	86
7059	78	NA	Metal Ages	Diaphysis	0.710486	0.000009	135
7060	79	NA	Metal Ages	Diaphysis	0.710869	0.000011	114
6133	81	NA	Metal Ages	Diaphysis	0.711740	0.000011	107
7061	82	NA	Metal Ages	Diaphysis	0.709462	0.000009	107
7062	83	KIA-34893 (2435±35)*	Early Iron Age	Diaphysis	0.709455	0.000009	83
7063	84	IRPA-476 (2430±50, charcoal)*	Early Iron Age	Diaphysis	0.709302	0.000009	132
7064	85	NA	Metal Ages	Diaphysis	0.709174	0.000011	104
7065	86	KIA-34180 (2390±30)*	Metal Ages	Diaphysis	0.709452	0.000012	125
-	87	IRPA-477 (2410±55, charcoal)*	Early Iron Age	NA	NA	NA	NA
7066	88	NA	Metal Ages	Diaphysis	0.709989	0.000014	132
6134	89	NA	Metal Ages	Diaphysis	0.709559	0.000007	122
7067	90	KIA-36921 (2505±30)*	Early Iron Age	Diaphysis	0.709722	0.000009	134
7068	91	KIA-34179 (2400±30)*	Early Iron Age	Diaphysis	0.709444	0.000011	114
7069	93	KIA-36922 (2520±30)*	Early Iron Age	Diaphysis	0.710526	0.000011	77
7070	94	NA	Metal Ages	Diaphysis	0.709813	0.000012	165
7071	95	NA	Metal Ages	Diaphysis	0.709152	0.000009	96
7072	96	NA	Metal Ages	Diaphysis	0.709635	0.000010	121
7073	97	NA	Early Iron Age	Diaphysis	0.709064	0.000009	149
7074	98	NA	Metal Ages	Diaphysis	0.709810	0.000008	177
7075	99	KIA-36923 (2665±40)*	Late Bronze Age	Diaphysis	0.709583	0.000011	80
7076	100	KIA-36926 (2785±30)*	Late Bronze Age	Diaphysis	0.709574	0.000012	130
7077	101	NA	Metal Ages	Diaphysis	0.709418	0.000008	87

7078	102	NA	Late Bronze Age	Diaphysis	0.709472	0.000009	89
7079	103	KIA-36927 (2775±30)*	Late Bronze Age	Diaphysis	0.709779	0.000009	92
6135	104	NA	Late Bronze Age	Diaphysis	0.709178	0.000018	107
7080	105	NA	Metal Ages	Diaphysis	0.710146	0.000008	99
6136	106	NA	Metal Ages	Diaphysis	0.709297	0.000008	113
7084	98-209	NA	Roman Period	Cranium	0.709461	0.000010	203
7086	98-218	NA	Roman Period	NA	0.709431	0.000009	186
7087	98-226	NA	Roman Period	NA	0.709578	0.000009	183
7088	98-232	NA	Roman Period	Diaphysis	0.709641	0.000008	168
7085	98-234	NA	Roman Period	Cranium	0.709347	0.000010	136
7089	98-236	NA	Roman Period	Diaphysis	0.709469	0.000011	133
7083	98-63	NA	Roman Period	Diaphysis	0.709320	0.000009	208
7209	ge<br>(AMUG_00498<br>_0032)	NA	Roman Period	NA	0.709541	0.000006	159
7210	gh<br>(AMUG_00501<br>_0002)	NA	Roman Period	NA	0.709393	0.000007	208
7211	gp<br>(AMUG_00507<br>_0004)	NA	Roman Period	NA	0.709366	0.00001	237
7212	gv<br>(AMUG_00514<br>_0008)	NA	Roman Period	NA	0.709574	0.000006	208
7213	ge1<br>(AMUG_00518<br>_0007)	NA	Roman Period	NA	0.709380	0.000005	172
7216	gv1<br>(AMUG_00532<br>_0015)	NA	Roman Period	NA	0.709367	0.000008	205
7218	gy1<br>(AMUG_00535<br>_0003)	NA	Roman Period	NA	0.709330	0.000006	217
7219	gd2<br>(AMUG_00539<br>_008)	NA	Roman Period	NA	0.709302	0.000009	134
7220	gw2<br>(AMUG_00560<br>_0002)	NA	Roman Period	NA	0.709024	0.000008	212
7221	go3<br>(AMUG_00566<br>_0004)	NA	Roman Period	NA	0.709247	0.000007	144
7222	gs3<br>(AMUG_00569<br>_0005)	NA	Roman Period	NA	0.709289	0.000005	185
7223	gt3<br>(AMUG_00570<br>_0006)	NA	Roman Period	NA	0.709300	0.000007	188
7224	gu3<br>(AMUG_00571<br>_0003)	NA	Roman Period	NA	0.709262	0.000006	168
7225	gd4<br>(AMUG_00580<br>_0003)	NA	Roman Period	NA	0.709438	0.000005	172
7226	ge4<br>(AMUG_00581<br>_0002)	NA	Roman Period	NA	0.709374	0.000007	239

7227	gh4 (AMUG_00583 _0005)	NA	Roman Period	NA	0.709220	0.000007	174
7228	gi4 (AMUG_00585 _0003)	NA	Roman Period	NA	0.709750	0.000006	175
7230	gn4 (AMUG_00589 _0004)	NA	Roman Period	NA	0.709288	0.000007	234
7231	gw4 (AMUG_00598 _0002)	NA	Roman Period	NA	0.710035	0.000006	202
7090	gx-indiv1	RICH-29132 (1887±24)	Roman Period	Mandible	0.709412	0.000010	178
7091	gx-indiv2	RICH-29130 (1904±24)	Roman Period	Mandible	0.709779	0.000010	139
7092	gx-indiv3	RICH-29089 (1893±24)	Roman Period	Mandible	0.709413	0.000012	133
7093	gx-indiv4	NA	Roman Period	Mandible	0.709374	0.000009	198
7094	gx-indiv5	RICH-29085 (1861±24)	Roman Period	Mandible	0.709504	0.000006	105
7095	gx-indiv6	RICH-29131 (1891±25)	Roman Period	Mandible (juvenile)	0.709310	0.000008	138
7096	gx-indiv7	RICH-29127 (1898±26)	Roman Period	Mandible (juvenile)	0.709584	0.000008	137
7102	gx-random1	NA	Roman Period	Rib (juvenile)	0.709403	0.000011	162
7103	gx-random2	NA	Roman Period	Rib (juvenile)	0.709374	0.000009	153
7104	gx-random3	NA	Roman Period	Rib (juvenile)	0.709326	0.000011	144
7105	gx-random4	NA	Roman Period	Rib (juvenile)	0.709586	0.000009	115

Supplementary Table 3a.

Results of the selected cremated bone samples of Herstal, published by Sabaux et al (2021)[12]. (*[Sr] normalised to 40 wt% Ca).

Sample ID	Site	Grave number	^{14}C Age BP	Chronology	Bone type	$^{87}Sr/^{86}Sr$	2σ	[Sr] (ppm)*
6082	Herstal	1	NA	Late Bronze Age	Diaphysis	0.712696	0.000007	91
4230	Herstal	2A	RICH-28688 (2896±24)	Middle-Late Bronze Age	Diaphysis	0.712379	0.000010	95
8063	Herstal	3A	NA	Late Bronze Age	Diaphysis	0.712402	0.000018	89
8066	Herstal	4A	RICH-29015 (2806±24)	Late Bronze Age	Diaphysis	0.713393	0.000011	121
6096	Herstal	5	NA	Late Bronze Age	Diaphysis	0.711724	0.000007	114
4236	Herstal	6A	RICH-28697 (2730±26)	Late Bronze Age	Diaphysis	0.711697	0.000011	98
8006	Herstal	7A	NA	Late Bronze Age	Diaphysis	0.712885	0.000006	121
6090	Herstal	8	NA	Late Bronze Age	Diaphysis	0.712324	0.000007	80
4239	Herstal	9	RICH-28709 (2770±26)	Late Bronze Age	Diaphysis	0.711600	0.000014	111
8054	Herstal	10	NA	Late Bronze Age	Diaphysis	0.713290	0.000020	89
6092	Herstal	11	NA	Late Bronze Age	Diaphysis	0.712188	0.000009	112
8009	Herstal	12	NA	Late Bronze Age	Diaphysis	0.712538	0.000008	103
8081	Herstal	13	NA	Late Bronze Age	Diaphysis	0.711686	0.000015	63
8082	Herstal	14	NA	Late Bronze Age	Diaphysis	0.713468	0.000010	99
6094	Herstal	15	NA	Late Bronze Age	Diaphysis	0.711714	0.000011	78
8011	Herstal	16	NA	Late Bronze Age	Diaphysis	0.711715	0.000010	110
8004	Herstal	17	NA	Late Bronze Age	Diaphysis	0.712088	0.000008	87
6097	Herstal	18	NA	Late Bronze Age	Diaphysis	0.712380	0.000008	95
8088	Herstal	19	RICH-29014 (2754±25)	Late Bronze Age	Diaphysis	0.710911	0.000013	93
6098	Herstal	20	NA	Late Bronze Age	Diaphysis	0.712505	0.000009	81
6093	Herstal	21	NA	Late Bronze Age	Diaphysis	0.712553	0.000008	104

Supplementary Table 3b.

Results of the cremated bone samples of Blicquy and Fize-le-Marsal. Radiocarbon dating and Sr analysis of Fize-le-Marsal are not performed on the same bone sample, but on associated fragments from the same burial (*[Sr] normalised to 40 wt% Ca).

Sample ID	Site	Grave number	14C Age BP	Chronology	Bone type	87Sr/86Sr	2σ	[Sr] (ppm)*
5049	Blicquy	F9	NA	Metal Ages	Diaphysis	0.710408	0.000022	111
5054	Blicquy	F13-27	NA	Middle-Late Bronze Age	Diaphysis	0.710347	0.000018	119
5057	Blicquy	F45	NA	Middle-Late Bronze Age	Cranium	0.709803	0.000009	76
5059	Blicquy	F68	NA	Middle-Late Bronze Age	Diaphysis	0.7103374	0.000011	69
5065	Blicquy	F72	NA	Middle-Late Bronze Age	Diaphysis	0.710148	0.000008	67
5068	Blicquy	F79	NA	Middle-Late Bronze Age	Diaphysis	0.710364	0.000011	49
5071	Blicquy	F83	NA	Early Iron Age	Diaphysis	0.7106303	0.000012	53
5079	Blicquy	F163	NA	Metal Ages	Diaphysis	0.7109444	0.000008	91
5082	Blicquy	F178	NA	Middle-Late Bronze Age	Diaphysis	0.710473	0.000019	92
1336	Blicquy	109	RICH-29544 (1954±25)	Roman period	Diaphysis	0.7098601	0.000009	110
1334	Blicquy	210	RICH-29639 (1910±27)	Roman period	Diaphysis	0.7100585	0.000010	160
1335	Blicquy	214	RICH-29638 (1905±28)	Roman period	Diaphysis	0.7095267	0.000009	118
1332	Blicquy	318	RICH-29511 (1827±25)	Roman period	Diaphysis	0.7095292	0.000010	123
1352	Blicquy	369	RICH-29878 (1961±25)	Roman period	Diaphysis	0.709649	0.000007	137
6067	Fize-le-Marsal	T01	NA	Roman period	Diaphysis	0.709354	0.000009	183
6060	Fize-le-Marsal	T02	NA	Roman period	Diaphysis	0.709596	0.000009	158
6062	Fize-le-Marsal	T04	NA	Roman period	Diaphysis	0.710807	0.000007	142
6063	Fize-le-Marsal	T06	NA	Roman period	Diaphysis	0.709559	0.000007	151
6070	Fize-le-Marsal	T10	NA	Roman period	Diaphysis	0.710767	0.000007	178

6071	Fize-le-Marsal	T12	NA	Roman period	Diaphysis	0.709862	0.000007	120
9036	Fize-le-Marsal	T13	NA	Roman period	Diaphysis	0.709716	0.000011	165
6072	Fize-le-Marsal	T14	NA	Roman period	Cranium	0.709508	0.000009	167
9038	Fize-le-Marsal	T15	NA	Roman period	Diaphysis	0.709539	0.000010	138
6073	Fize-le-Marsal	T16	NA	Roman period	Diaphysis	0.709465	0.000009	164
6075	Fize-le-Marsal	T17	NA	Roman period	Diaphysis	0.711886	0.000008	128
6077	Fize-le-Marsal	T18	NA	Roman period	Diaphysis	0.709958	0.000008	124
9020	Fize-le-Marsal	T19*	RICH-26967 (1772±24)	Roman period	Diaphysis	0.709889	0.000024	170
6079	Fize-le-Marsal	T20	NA	Roman period	Diaphysis	0.710659	0.000007	154
9018	Fize-le-Marsal	T21*	RICH-26968 (1910±21)	Roman period	Diaphysis	0.710115	0.000016	194
9023	Fize-le-Marsal	T22*	RICH-27012 (1837±23)	Roman period	Diaphysis	0.709536	0.000016	262
6080	Fize-le-Marsal	T23	NA	Roman period	Diaphysis	0.709897	0.000010	134
9026	Fize-le-Marsal	T24*	RICH-27014 (1861±23)	Roman period	Diaphysis	0.709652	0.000022	198

Supplementary Table 4.
Overview table of the plant samples measured around the site of Destelbergen.

Sample location	Latitude	Longitude	Geological information	Sample name	Plant type	$^{87}Sr/^{86}Sr$	2σ	$^{87}Sr/^{86}Sr$ median
Dest_1	51.05378	3.79685	Quaternary clastic sediment/Alluvion	Dest_1G	grass	0.710704	0.000008	0.7104
				Dest_1S	shrub	0.710398	0.000012	
				Dest_1T	tree	0.709611	0.000008	
Dest_2	51.05891	3.77381	Predominantly Eocene clay with subordinate siltstone	Dest_2G	grass	0.710031	0.000012	0.7102
				Dest_2S	shrub	0.710572	0.000011	
				Dest_2T	tree	0.710248	0.000009	
Be_83	51.04755	3.83038	Quaternary clastic sediment/Alluvion	Be_83G	grass	0.709974	0.000009	0.7095
				Be_83S	shrub	0.709543	0.000010	
				Be_83T	tree	0.709537	0.000009	
Be_157	51.07028	3.65307	Predominantly Eocene clay with subordinate siltstone	Be_157G	grass	0.710388	0.000010	0.7095
				Be_157S	shrub	0.709496	0.000012	
				Be_157T	tree	0.709199	0.000012	
Dest_3	51.05378	3.75740	Predominantly Eocene clay with subordinate siltstone	Dest_3G	grass	0.710564	0.000012	0.7101
				Dest_3S	shrub	0.710124	0.000010	
				Dest_3T	tree	0.709981	0.000011	
BG_R	51.06388	3.68447	Quaternary clastic sediment/Alluvion	BG_RG	grass	0.709873	0.000009	0.7093
				BG_RS	shrub	0.709255	0.000012	
				BG_RT	tree	0.709219	0.000009	

Supplementary materials references

1. Capuzzo, G. *et al.* Cremation vs. inhumation: modeling cultural changes in funerary practices from the Mesolithic to the Middle Ages in Belgium using Kernel Density Analysis on ^{14}C data. *Radiocarbon* **62**, 1809–1832 (2020).
2. De Reu, J., De Mulder, G., Van Strydonck, M., Boudin, M. & Bourgeois, J. ^{14}C dates and spatial statistics: Modeling intrasite spatial dynamics of urnfield cemeteries in Belgium using case study of Destelbergen cemetery. *Radiocarbon* **54**, 635–648 (2012).
3. De Clercq, W. Roman rural settlements in Flanders. Perspectives on a 'non-villa' landscape in extrema Galliarum. in *Villa Landscapes in the Roman North. Economy, culture and lifestyles* (eds. Roymans, N. & Derks, T.) 235–258 (Amsterdam University Press, 2011).
4. De Laet, S. J. *et al. Oudheidkundige opgravingen en vondsten in Oost-Vlaanderen. Vijfde reeks.* (1970).
5. De Logi, A. & Dalle, S. *Destelbergen – Panhuisstraat archeologisch onderzoek – 2011*. (2013).
6. Bongers, T. Connectivity in the Scheldt Basin : The role of the river Scheldt in the Roman-era transport network 1. *Digit. Class. Online* **6**, 33–58 (2020).
7. Kiden, P. De evolutie van de Beneden-Schelde in België en zuidwest-Nederland na de laatste ijstijd. *Belgeo* 279–294 (2006).
8. Jongepier, I. Drowned but not deserted. Interactions between social and ecological processes of estuarine landscapes after flooding. Test-case: the Waasland polders on the left-bank of the river Scheldt (sixteenth to nineteenth centuries). (University of Antwerp, 2015).
9. Scheidel, W. Building ORBIS: Historical evidence. https://orbis.stanford.edu/#rivertransport.
10. Mathys, M. The Quaternary geological evolution of the Belgian Continental Shelf, southern North Sea. (Ghent University, 2009).
11. Alenus-Lecerf, J. Sondages dans un champ d'urnes à Herstal. *Archaeol. Belgica* **157**, 5–41 (1974).
12. Sabaux, C. *et al.* Multi-proxy analyses reveal regional cremation practices and social status at the Late Bronze Age site of Herstal, Belgium. *J. Archaeol. Sci.* **132**, 105437 (2021).
13. Destexhe, G. La nécropole de Crisnée/Fize- le-Marsal (province de Liège). in *Du bûcher à la tombe. Les nécropoles à incinération gallo-romaines en Wallonie* (eds. Hanut, H. & Henrotay, D.) 140–145 (Institut du Patrimoine wallon, 2014).
14. Leclercq, W. Les nécropoles de l'âge du Bronze Final entre les basins de l'Escaut et de la Meuse moyenne: approche chronologique et culturelle de leur occupation. in *Des espaces aux esprits: l'organisation de la mort aux âges des Métaux dans le nord-ouest de l'Europe* (eds. Cahen-Delhaye, A. & De Mulder, G.) 15–28 (Institut du Patrimoine Wallon, 2014).
15. De Mulder, G. *et al.* Re-evaluation of the Late Bronze Age and Early Iron Age chronology of the Western Belgian urnfields based on 14C dating of cremated bones. *Radiocarbon* **49**, 499–514 (2007).
16. Stamataki, E. *et al.* Is it hot enough? A multi-proxy approach shows variations in cremation conditions during the Metal Ages in Belgium. *J. Archaeol. Sci.* **136**, (2021).
17. Gillet, E., Paridaens, N. & Demarez, L. Le sanctuaire de Blicquy - Ville d'Anderlecht (prov. Hainaut, Belgique). Itinéraire d'une topographie religieuse dans la cité des Nerviens. in *Sanctuaires, pratiques cultuelles et territoires civiques dans l'occident Romain* (eds. Dondin-Payre, M. & Raepsaet-Charlier, M.-T.) 181–215 (2006).
18. Van Doorselaer, A. *Repertorium van de begraafplaatsen uit de Romeinse tijd in Noord-Gallië / Répertoire des nécropoles d'époque Romaine en Gaule Septentrionale / Repertorium der Römerzeitlichen Gräber in Nord-Gallien*. (1964).
19. De Laet, S. J., Van Doorselaer, A., Spitaels, P. & Thoen, H. *La nécropole Gallo-Romaine de Blicquy (Hainaut, Belgique)*. (De Tempel, 1972).
20. Veselka, B. *et al.* Divergence, diet, and disease: The identification of group identity, landscape use, health, and mobility in the fifth- to sixth-century AD burial community of Echt, the Netherlands. *Archaeol. Anthropol. Sci.* (2021).
21. De Mulder, G. Funeraire rituelen in het Scheldebekken tijdens de late bronstijd en de vroege

ijzertijd. De grafvelden in hun maatschappelijke en sociale context. (Ghent University, 2011).
22. Acsadi, G. T. & Nemeskéri, J. *History of human life span and mortality*. (Akademiai Kiado, 1970).
23. Janssens, P. A. *Antropologisch onderzoek van de gecremeerde beenderresten uit het U.V.K.-grafveld van Destelbergen-Eenbeekeinde*.
24. Snoeck, C. A burning question: structural and isotopic analysis of cremated bone in archaeological contexts. (University of Oxford, 2014).
25. De Laet, S. J., Thoen, H. & Van Doorselaer, A. La tombe collective de la nécropole Gallo-Romaine de Destelbergen-Lez-Gand (Flandre Orientale). *Helinium* **10**, 3–30 (1970).
26. Ervynck, A., Decker, S. De, Dewilde, M., Quintelier, K. & Brion, M. *Omgaan met menselijke resten bij archeologisch onderzoek in Vlaanderen - versie 1. Afwegingskaders agentschap Onroerend Erfgoed* vol. 7 https://oar.onroerenderfgoed.be/publicaties/AKOE/7/AKOE007-001.pdf (2018).
27. De Laet, S. J., Thoen, H. & Bourgeois, J. *Les fouilles du séminaire d'archéologie de la Rijksuniversiteit te Gent à Destelbergen Eenbeekeinde (1960-1984) et l'histoire la plus ancienne de la région de Gent (Gand). I La période préhistorique*. (De Tempel, 1986).
28. De Vos, S. Het Gallo-Romeins grafveld van Destelbergen-Eenbeekeinde. Een studie van 60 brandrestengraven en 4 ustrina uit de opgravingscampagne van 1998. *VOBOV-Info* **59**, 17–26 (2004).
29. Cools, A. *Inpakken, een kunst. Het verpakken van archeologische vondsten*. https://oar.onroerenderfgoed.be/publicaties/VIOH/1/VIOH001-001.pdf (2009).
30. De Laet, S. J. *et al.* De opgravingen te Destelbergen-Eenbeekeinde. in *Oudheidkundige opgravingen en vondsten in Oost-Vlaanderen VIII* 8–63 (1978).
31. Cherretté, B. & Dhaeze, W. Archeologisch noodonderzoek op de site Destelbergen-Eenbeekeinde (2001-2002). *Romeinendag* 9–11 (2003).
32. Evans, J. A., Chenery, C. A. & Montgomery, J. A summary of strontium and oxygen isotope variation in archaeological human tooth enamel excavated from Britain. *J. Anal. At. Spectrom.* **27**, 754–764 (2012).
33. De Mulder, G., Van Strydonck, M., Boudin, M. & Deweirdt, E. Een voorlopig overzicht van de ^{14}C-resultaten op gecremeerd bot en houtskool van het urnengrafveld te Destelbergen (provincie Oost-Vlaanderen, België). *Lunula, Archaeol. protohistorica* **17**, 65–71 (2009).
